# A Universal Approach to Mie Scatter Correction in FTIR Analysis of Microsized Samples

**DOI:** 10.1021/acsomega.5c07884

**Published:** 2025-12-10

**Authors:** Uladzislau Blazhko, Eirik Magnussen, Johanne Solheim, Simona Dzurendova, Volha Shapaval, Achim Kohler

**Affiliations:** Faculty of Science and Technology, 56625 Norwegian University of Life Sciences, 1432 Ås, Norway

## Abstract

The inverse Mie scattering problem (IMSP) is extensively studied across various scientific fields due to its relevance in characterizing particles through light scattering. In infrared microspectroscopy, effects of Mie-like scattering significantly bias absorbance spectra complicating studies of microscopic objects. A general solution of the IMSP would allow to restore chemical information on a sample without any preknowledge about the absorption properties of the samples. Herein, we report on a deep-learning based Mie scattering correction that can be universally applied to an infrared spectrum of any sample kind. We compared the novel method with other approaches that were developed for and are valid only for specific types of samples. For validation we use a wide range of real-world validation samples such as microplastic beads, lung cells, and filamentous fungi that were measured in various spectroscopic setups, including a single detector and a focal plane array detector. Finally, we shed light on the uniqueness of the IMSP for spectral data. We find that in the vicinity of the true solution of the IMSP, all solutions are getting a characteristic distortion that cannot be typically observed in spectra, and therefore can be effectively sieved out by the suggested approach. The novel approach allows for the first time the retrieval of infrared spectra for infrared microspectroscopic studies in a quick way without requiring preknowledge about absorption properties of the samples investigated. Our approach offers a transferable framework for solving inverse Mie scattering problems across diverse scientific fields.

## Graphical abstract

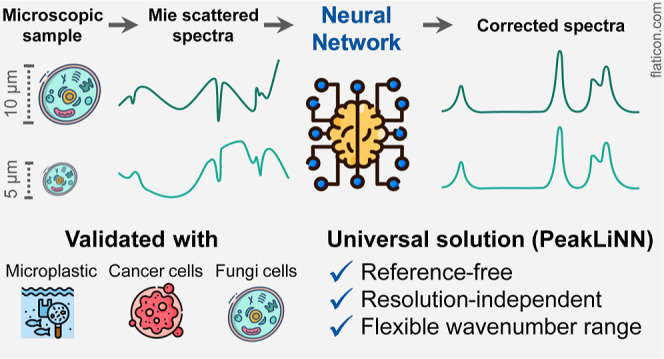

## Introduction

Midinfrared (MID-IR) microspectroscopy is a powerful and indispensable analytical technique delivering spatially resolved chemical insights on a microscopic level. This capability is crucial for advancing our understanding of complex materials and biological systems.^1–4^ Yet, beneath its esteemed utility lies a fascinating challenge: two chemically identical samples can yield strikingly different absorbance spectra simply due to variations in their size or shape.^5–7^ As illustrated in the Supporting Information (Movie 1), this discrepancy arises from the intricate interplay between absorption and scattering of infrared radiation. While a sample is expected to absorb infrared light, it may also scatter light in multiple directions, complicating the interpretation of whether radiation loss is due to absorption or scattering (Movie 1). Consequently, scattering introduces a bias in the absorbance signature, adding an intriguing layer to the analysis that researchers must navigate to exploit the full potential of MID-IR microspectroscopy.

Highly nonlinear scattering effects emerge prominently when the wavelength of light is comparable to the size of the sample.^8^ Given that wavelengths in the MID-IR range span microscopic dimensions (2.5 to 25 μm), infrared microscopic measurements of biological cells exhibit pronounced scattering effects.^5,9^ This phenomenon is observed across various biological entities, including human cells,^9^ tissues,^10^ bacteria,^11^ yeasts,^12^ and pollens,^6^ all demonstrating significant scattering. These scattering effects pose a substantial challenge to the chemical interpretation of the measured spectra.^5,9^ As a result, spectra of cells and tissues may reflect not only the chemical composition of the sample but also its morphological characteristics, such as size and shape.^13^ Although scattering effects have the potential to provide insights into the morphology of the sample,^7,13^ they significantly complicate the chemical analysis of microscopic samples due to the difficulty in isolating pure scattering features.

Mie theory has been increasingly utilized in recent years to describe the scattering and absorption properties of biological cells and tissues.^14–17^ It provides a comprehensive framework for understanding the behavior of light as it interacts with homogeneous spherical particles. By taking into account the size and optical characteristics (complex refractive index) of the sphere, along with the wavelength of the electromagnetic radiation, Mie theory can accurately predict the scattering and absorption properties of an absorbing sphere. Numerous studies have demonstrated that Mie theory effectively models biological cells and tissues, even when their shapes deviate significantly from spherical forms.^18–21^ This suggests that Mie theory serves as a reliable first-order approximation for practical applications.

The challenge of extracting a pure absorbance spectrum from its scattered counterpart is known in physics as an inverse scattering problem (ISP). ISPs are prevalent across various fields, from medical imaging to seismology, and provide noninvasive insights into hidden or otherwise inaccessible information.^22–24^ Theoretically, the inverse Mie scattering problem has been proven to have a unique solution when the intensity and phase of the scattered radiation are known across all solid angles.^25^ However, in infrared microspectroscopy, measurements are confined to the direction of the detector, within a very narrow solid angle. Consequently, the ISP in infrared microscopy is generally expected to be nonunique. Inverse problems, in contrast to forward problems, are typically ill-posed because the scattered field does not correspond to a unique scattering scenario. Even with comprehensive measurements of the scattered field, the precise physical and chemical properties of the scatterer remain elusive.^26,27^ To address this challenge, we proposed previously a strategic approach to eliminate irrelevant solutions of the ISP, thereby identifying a single relevant solution through the introduction of conditioning.^13,28–30^ These conditions can be broadly categorized into chemical and morphological conditioning. Chemical conditioning involves leveraging known chemical properties or constraints of the system under investigation. For example, when studying a fungal sample, the reference spectrum of the fungus, which contains characteristic spectral features, can be used to filter out dissimilar spectra (solutions). Morphological conditioning utilizes information about the physical shape, structure, or spatial characteristics of the sample. By incorporating such morphological details, we can further narrow down the possible solutions to those that are consistent with the observed physical attributes of the sample. Utilizing Mie theory allows for the refinement of the solution space to include only those solutions that align with the inverse Mie scattering problem. By applying these conditioning strategies, we aim to refine the solution space and achieve a more accurate and unique determination of the scatterer’s properties.

The state-of-the-art solution for Mie scatter correction, known as the Mie-extinction extended multiplicative signal correction (ME-EMSC) method,^29^ employs both chemical and morphological conditioning. The morphology of the sample is conditioned to the shape of a sphere, while chemical conditioning is achieved through the use of a reference spectrum. The reference spectrum provides the initial solution for the ME-EMSC algorithm that is then iteratively improved. It guides the algorithm to find a local minimum near the given reference spectrum, which can be considered as conditioning with respect to chemistry. Therefore, the reference spectrum must be chosen such that its chemical signature is not too different from the chemical information contained in the spectrum to be corrected. For instance, when dealing with spectra of a specific cell type, reference spectra of similar cell types can be used. This need for chemical conditioning in the ME-EMSC algorithm necessitates that operators must obtain a tailored reference spectrum for each specific type of cell, adapting them to the given problem. Another notable iterative Mie scatter correction method is conditioned to homogeneous spheres.^31^ However, this algorithm requires prior knowledge of the cell size, which is not always practical. Although iterative methods like ME-EMSC and its variants have been employed for several years, their major drawbacks are their validity for very specific situations and the substantial computation time required.

Recently, several deep learning methods have been developed for Mie scatter correction, where neural networks are trained on data sets containing both measured spectra and ME-EMSC corrected spectra.^30,32^ Raulf et al. trained a fully connected contractive stacked autoencoder to predict a corrected spectrum as per the RMieS method (a variant of ME-EMSC).^32^ Magnussen et al. trained a convolutional descattering autoencoder to predict a corrected spectrum based on the ME-EMSC method.^30^ A noteworthy outcome of this work is that the training set included spectra corrected using different reference spectra, enabling the neural network to implicitly select an appropriate reference spectrum for the correction task. An impressive achievement was realized when a neural network, conditioned to the morphology of two-layered concentric spheres and a chemical subspace of diverse biological systems, successfully predicted the absorbance spectrum of both the cell wall and cell interior, as well as their radii, from a single scattered spectrum.^13^ Guo et al. trained a neural network (1D U-Net) to predict a simulated pure absorbance spectrum of a poly­(methyl methacrylate) (PMMA) sphere from measured scatter-distorted simulated Mie-scattered spectra of PMMA spheres.^33^ In Guo et al.‘s work, the chemical conditioning was very narrow, as the model contained the chemical signature of a pure absorbance spectrum of a PMMA sphere.

In this work, we aim, for the first time, to develop a universal approach for scatter correction of microsized samples that is applicable to any type of microparticle (e.g., cells, tissues, and microplastics) and does not require chemical conditioning. We present a deep learning-based method that can be used right off-the-shelf to correct a wide range of spectra from various sources. Furthermore, we explore the interplay between chemical and morphological conditioning by comparing universal and chemistry-aware approaches. Finally, through a numerical analysis of the inverse Mie scattering problem, we shed light on the circumstances under which scatter correction can be performed without chemical conditioning. This represents a significant advancement in the field, paving the way for more efficient and versatile scatter correction techniques.

## Experimental Section

### Neural Networks for Inverse Scatter Models

We present two innovative neural network models for Mie scatter correction (Figure 1): the universal Peak Limited Neural Network (PeakLiNN) and the Chemistry Limited Neural Network (ChemLiNN), both of which incorporate the principles of Mie theory. These models follow the physics-informed neural network (PINN) framework, where physical principles are integrated into the learning process. While physics-informed neural networks typically incorporate physics knowledge through a loss function,^34,35^ they have been shown to struggle with learning complex physics setups.^36^ The Mie scatter formalism is a complex framework that is expected to represent a similarly challenging scenario owing to its highly nonlinear formalism.^37^ Therefore, we incorporated physics knowledge about Mie scattering into the PeakLiNN and ChemLiNN models through physics-simulated data (see Supporting Information for details). When sufficient training data are available, a purely data-driven approach can perform comparably to incorporating partial differential equations in the loss function.^38^ Both neural networks share the same fully convolutional encoder-decoder architecture (Figure S1 in Supporting Information) but differ in the types of spectra they were trained on. The first approach, PeakLiNN, limits the training set to spectra representing all conceivable chemical compositions, characterized by a combination of Gauss–Lorentzian peak profiles with varying positions, heights, and widths—hence the name peak-limited approach. The second approach, ChemLiNN, restricts the training set to spectra representing diverse chemical fingerprints of particular types of samples. Thus, the ChemLiNN approach specializes in correcting spectra of specific classes of samples, while the PeakLiNN approach aims to learn a correction that can be generally applied to any sample. It is important to highlight that both approaches provide a form of conditioning, which contributes to the potential uniqueness of solutions. However, the ChemLiNN approach imposes a much stricter conditioning compared to the PeakLiNN approach. This distinction underscores the versatility and applicability of PeakLiNN across a broader range of sample types, while ChemLiNN offers enhanced specificity and precision for particular sample classes. The architecture of these neural networks is detailed in the Supporting Information.

**Figure 1:**
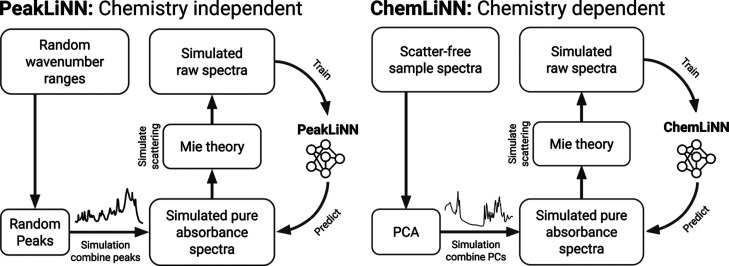
Proposed Mie scatter correction methods. The universal approach (PeakLiNN, peak limited neural network) simulates a training set generating pure absorbance spectra by combining several random Lorenz–Gaussian profiles at random wavenumber ranges. Then it simulates Mie scattered spectra using the Mie theory employing various geometric and optical parameters. Finally, it trains a convolutional neural network (CNN) to transform the simulated Mie scattered spectra into the simulated pure absorbance spectra. The chemistry-aware approach (ChemLiNN, chemistry limited neural network) is different only in the simulation of pure absorbance spectra that should remind spectra of samples of interest. It starts from a data set of scatter-free spectra, from which it learns scatter-free chemical features using principal components (PCs) obtained by principal components analysis (PCA). Then it randomly combines the learned PCs to produce pure absorbance spectra representing samples from the data set. Thus, the ChemLiNN method is conditioned to a particular chemical composition and therefore can only be applied to spectra of samples that are chemically similar to the scatter-free spectra data set, while the PeakLiNN method is conditioned only by the physics of the Mie scatter process and can therefore be generally applied to any spectrum. Neural network icon: Flaticon.com.

### Spectra Simulation

The simulation of the training set consisted of two steps. First, pure absorbance spectra were generated. Second, Mie scattering was simulated for each pure absorbance spectrum. The neural networks were then trained to reconstruct the pure absorbance spectrum from the corresponding scattered spectrum. A new set of spectra was generated for each training batch. The two simulationn steps are described in detail below.

### Pure Absorbance Spectra Simulation

For the PeakLiNN approach we first generated a dynamic range of wavenumbers containing 1408 points. The starting point varied between 400 to 3300 cm^–1^ and the step size (i.e., spectral resolution) ranged from 0.5 to 3 cm^–1^. As a result, the full wavenumber range could extend up to approximately 6000 cm^–1^. To avoid aligning wavenumbers perfectly on a regular grid, we added small, normally distributed noise depending on the resolution of the generated wavenumber range (mean = 0, std = resolution × 0.001) to each point. Within this range, we randomly generated a set of Lorentz–Gaussian profiles, as described by Stancik and Brauns.^39^ Each profile was a linear combination of asymmetric Lorentzian and Gaussian shapes with random amplitude, position, width, and skew. We distinguished between “sharp” (full width at half-maximum, fwhm <100 cm^–1^) and “wide” (fwhm >100 cm^–1^) profiles. Asymmetry was present only in the wide profiles, while the sharp profiles exhibited zero skew. For each spectrum, we included up to 15 wide and up to 60 sharp peaks, reflecting the typical composition of real spectra, which tend to contain more narrow features. This synthetic data set exposed the PeakLiNN model to a broad range of spectral patterns, resolutions, and ranges. The intention was to enable training on spectra that reflect the diversity encountered across different spectrometers and measurement conditions.

While ChemLiNN is a general approach to introduce chemical conditioning to the neural network, in paper we demonstrated it with hyperspectral images of filamentous fungus *Mucor circinelloides*. To inform the ChemLiNN model about chemical variability of the filamentous fungus, we represented the underlying chemistry by a linear combination of predefined components. The components were derived in an unsupervised manner using principal components analysis (PCA) of transmission spectra obtained with the Fourier transform infrared spectroscopy (FTIR) on homogenized filamentous fungi grown under different conditions.^40^ Homogenization of the samples allowed to obtain spectra without Mie scattering effects, while varying growing conditions introduced diversity in spectra implicitly containing information about presence of various chemical components in filamentous fungi such as, for example, chitin and lipids. Notably, the obtained chemical diversity in homogenized samples allowed to describe spatial morphological variation in hyperspectral images of the filamentous fungi. Before the PCA analysis, the FTIR spectra were preprocessed by extended multiplicative signal correction (EMSC) with second derivative polynomial order.^41^ The first seven principal components describing 99% of variability in the measured spectra of homogenized samples were used to represent chemical diversity of the filamentous fungi. Mixing those principal components in different proportions and adding them to the mean spectrum we simulated pure absorbance spectra of filamentous fungi. The coefficients were generated in the range from minimal to maximal score values. Since principal components by default were vector normalized, i.e. had the same area under the curve, we renormalized them to the mean peak-to-peak value to mix them based on peak heights rather than on area under the curve. The use of the exaggerated scores values and the peak-to-peak normalized components helped to generate spectra with more pronounced variation of different components, and therefore to better simulate possible heterogeneous compositions (Figure S3 in Supporting Information).

### Scattering Simulation

Each simulated pure absorbance spectrum was used to generate the corresponding scattered spectrum. These simulated scattered spectra represent what could theoretically be measured in an actual experimental setup. The scattering effects were simulated using Mie theory^37^ taking into account numerical aperture.^15^ In an attempt to go beyond the rigorous simulation of scattering by an ideal spherical particle we linearly perturbed several theoretical variables such as effective optical path and constant baseline of imaginary refractive index in an empirical manner. Algorithms for Mie scattering simulation were developed in-house using Cython^42^ based on Matzler’s Matlab implementation.^43^ Further details are provided in the Supporting Information.

### Real-World Validation Data

To validate our correction approaches under realistic conditions, we tested them on infrared data sets of poly­(methyl methacrylate) PMMA microplastic beads, human lung cancer cells, and filamentous fungi. The selected data sets encompass a wide range of morphological forms, including perfect spheres, nonuniform spheres with internal substructures, and thread-like networks of interconnected rods and irregular spheres. They also cover both homogeneous and heterogeneous chemical compositions. PMMA microspheres with a diameter of approximately 5.5 μm have uniform, spherical shape and known homogeneous composition. These idealized properties fulfill the assumptions of the Mie theory; consequently, the measured scattering features closely resemble those predicted by the theory. This makes PMMA spheres a standard reference for Mie scattering modeling in infrared (IR) microscopy and thus represents a simple test case for the correction. We measured spectra using both synchrotron and globar sources with single-element and focal plane array (FPA) detectors, providing well-controlled conditions for baseline comparison. Human lung cancer cells are nonuniformly spherical and chemically heterogeneous biological samples. Their structural and chemical heterogeneity violates the assumptions of the Mie theory, resulting in a Mie-like scattering features. This makes the correction more challenging and thus represents a moderate-complexity test case. These were measured at the European synchrotron radiation facility (ESRF) synchrotron using a single-element detector.^28^ PeakLiNN correction results were compared to ME-EMSC as no clear ground truth exists. Filamentous fungi have a network-like morphology composed of interconnected rods and irregular spheres that differ in chemical composition and therefore they represent the most complex case for the correction. We used FTIR hyperspectral images of *M. circinelloides* grown under different nutrient conditions, captured with an FPA detector.^30^ ChemLiNN was evaluated only on the filamentous fungi data set, while PeakLiNN was evaluated across all three data sets.

## Results and Discussion

### The Universal Solution PeakLiNN

To demonstrate the versatility of the PeakLiNN model, we applied it to a broad selection of measured spectra with scattering features obtained from highly scattering microsamples, namely PMMA microplastic beads, lung cancer cells, and filamentous fungus. The PeakLiNN model worked well in most cases and was able to remove all the characteristic Mie scattering broad oscillations (Figure 2). Note that all corrected spectra slightly vary in wavenumber range and digital resolution. Those are automatically handled by the PeakLiNN model as it was trained on spectra simulated in various wavenumber ranges. Our PeakLiNN model, which we made publicly available (https://github.com/BioSpecNorway/peaklinn), can correct spectra with a wavenumber range within a region of 500 cm^–1^–6000 cm^–1^ and with a digital spacing ranging from 0.5 cm^–1^ to 3 cm^–1^. This covers most practically relevant cases in infrared spectroscopy of micro particles. In the following, we will consider the quality of the correction in more detail starting with nearly perfect homogeneous spherical microplastic beads and then considering more complex samples such as heterogeneous lung cancer cells and filamentous fungi with very complex morphologies.

**Figure 2:**
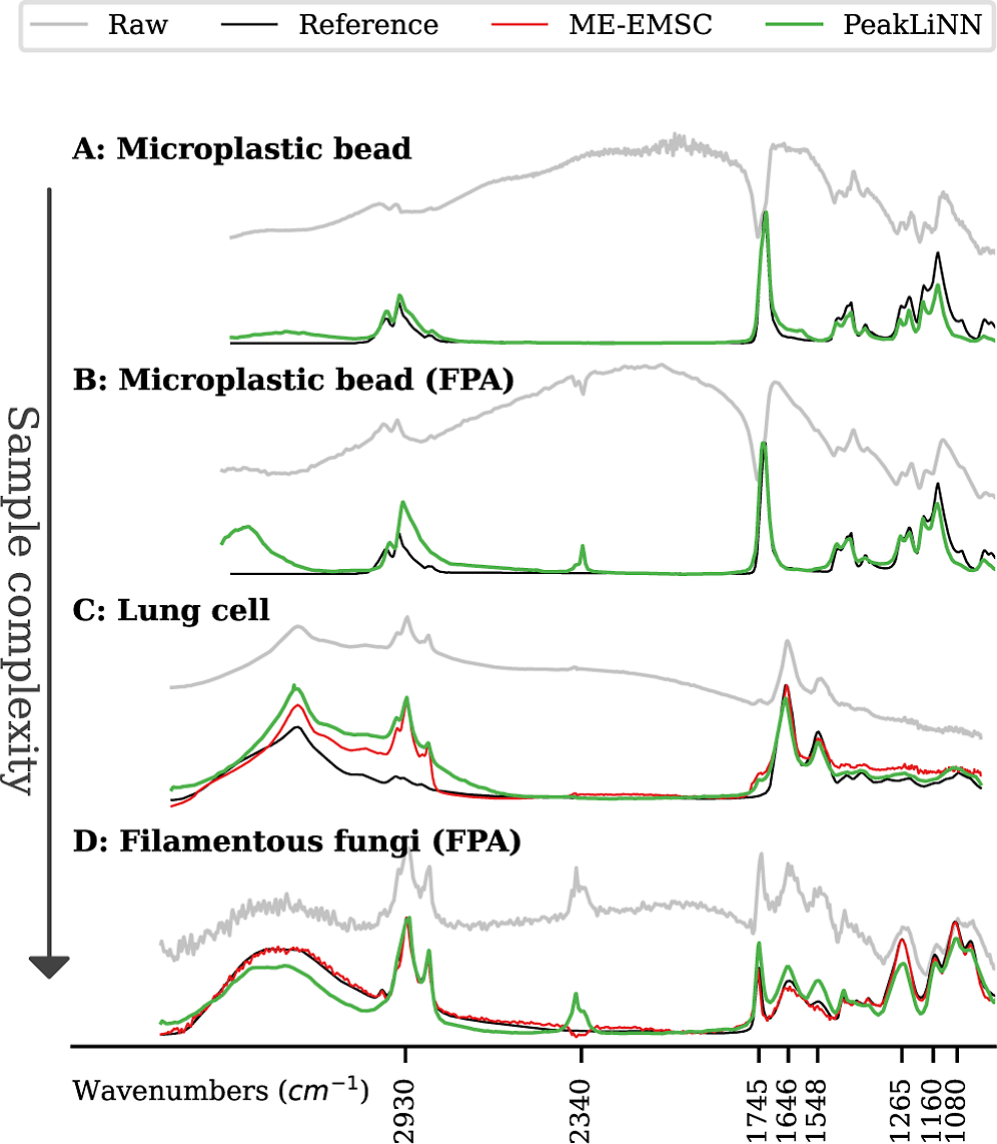
Spectra correction across various samples using PeakLiNN demonstrating its versatility in handling chemically diverse samples. Displayed are the raw scatter-distorted spectra (gray lines) and the PeakLiNN-corrected spectra (green lines) for: (A,B) microplastic (PMMA) bead of 2.75 μm radius that was measured by (A) an infrared microsope coupled to a synchrotron source and employing a single element detector and (B) an infrared microscope employing an FPA and a thermal infrared light source; (C) lung cell that was measured by an infrared microscope coupled to a synchrotron source and using a single element detector; (D) filamentous fungus that was measured by an Agilent FPA Microscope employing a thermal infrared source. For each sample, PeakLiNN corrections are compared against (A,B) a reference spectrum of PMMA material (black line) obtained by macroscopic infrared measurements of PMMA; (C, D) the state-of-the-art ME-EMSC method (red line) using a scatter-free reference spectrum (black dashed line).

Homogeneous microplastic PMMA spheres are often considered as a model object for Mie scattering in infrared microscopy. Microplastic beads of 2.75 μm radius were measured using a synchrotron light source coupled to a single element mercury cadmium telluride (MCT) detector (Figure 2A) and a globar radiation source coupled to a FPA MCT detector (Figure 2B). Comparing the corrected spectrum with the approximately pure spectrum of PMMA material, we can see that the PeakLiNN model correctly resolved all the absorption bands of PMMA material. For the single element detector spectrum obtained at a synchrotron facility, a better correction was achieved in the C–H stretching region around 3000 cm^–1^–2800 cm^–1^, while for the FPA data, a better correction was achieved in the region between 1500 cm^–1^–1000 cm^–1^. This may be due to diffraction blurring in the FPA detector—an effect not accounted for in the PeakLiNN model—which could have influenced the representation of scattering features.^44^

As samples with more complex morphologies, we consider lung cells measured by a single element detector (Figure 2C) and a hyperspectral image of a filamentous fungi measured by an FPA detector (Figure 2D). Due to their heterogeneity it was problematic to obtain a ground truth absorbance spectrum for comparison. Therefore, we compare the correction results by neural networks with results obtained by ME-EMSC corrected spectra. It is important to note that the ME-EMSC is not universal and needs to be adapted with a suitable reference spectrum to the samples under investigation, while the PeakLiNN model is a universal model. Despite the complex structure of the samples not aligning with the neural network’s assumption of homogeneous spherical particles, the PeakLiNN model still preserved all the primary chemical features. However, for the lung cell, the PeakLiNN method raised the left part of the spectrum. This does not happen for the ME-EMSC correction. It is unclear to what extent the ME-EMSC corrected spectrum represents the true absorbance spectrum of the lung cell and what the ground true is. Both the EMSC correction and the PeakLiNN’s correction may be influenced by the mismatch between the assumption of a single homogeneous sphere and potential double-scatter nature of cells, when the scattering may be caused by both the cell itself and its nucleus. In Figure 2D, the ME-EMSC solution did not recover two peaks, namely peaks at 2340 cm^–1^ and 1548 cm^–1^, which are present in the raw spectrum, but not in the corrected spectrum. Since the ME-EMSC is an iterative algorithm based on the Mie theory and multivariate modeling, the search of a solution is started in the neighborhood of a reference spectrum that is needed for initializing the algorithm and which is provided specifically for the cells under investigation. Therefore, the ME-EMSC may converge to a solution that is close to the reference spectrum which is not close to the underlying ground truth for the correction problem. The PeakLiNN model, however, recovered both peaks at 2340 cm^–1^ and 1548 cm^–1^, since the PeakLiNN model is not biased by a reference spectrum. It can be further seen, that the PeakLiNN method smoothed noise in the corrected spectrum. The PeakLiNN correction results generally in more smooth spectra than the ME-EMSC correction. The smoothing effect of the PeakLiNN correction can as well result in the removal of very small peaks, that the PeakLiNN identifies as noise. This can be seen in Figure 2D, where the PeakLiNN correction removed the tiny peak at 3010 cm^–1^, which is linked to =C–H stretching and represents the level of unsaturation in lipids.^45^ Implementing additional information on the underlying chemistry in the PeakLiNN model, may solve the problem that the PeakLiNN model has in separating between chemical information and noise with respect to, for example, position of peaks. However, implementing chemical information in the PeakLiNN model may reduce its general applicability. Further characteristics of the PeakLiNN model are elucidated in the following section comparing it with chemistry-aware models.

Generally, all the PeakLiNN-corrected spectra were meaningful and overall close to the profile of the reference spectrum. Even though the peak heights of the individual predicted peaks are in some cases not perfectly restored (the error will be elaborated later in the section on quantitative results), the PeakLiNN neural network shows a potential for reference-free correction of spectra. Reference-free correction of spectra may facilitate and speed up analysis of samples by infrared spectroscopy and, thus, extend its potential applicability in various domains. Most importantly, the PeakLiNN model is universally applicable and does not require the training of a new model for each new application as it would be required by the ChemLiNN model, neither, it requires the availability of reference spectra as it is required by the ME-EMSC model.

### Comparison of the Universal and Chemistry-Aware Approaches

To understand limitations of the universal model PeakLiNN, we compared it to two chemistry-aware models that were trained to correct spectra of the filamentous fungus *M. circinelloides*. The first model is a descattering autoencoder (DSAE)^30^ that was trained to predict the ME-EMSC corrected spectra of *M. circinelloides*. The second model is a neural network ChemLiNN. It was trained similarly to PeakLiNN with simulated scatter-distorted spectra according to Mie theory, but the scatter-free (pure) spectra were simulated based on real absorbance spectra of *M. circinelloides* representing a large variety of the chemical compositions of this fungus. The pure spectra were sampled from a principal component analysis (PCA) subspace, a process, which can be considered as augmentation of the original set of pure spectra. Thus, in contrast to chemically universal PeakLiNN solution, ChemLiNN is conditioned to spectra representing the chemical compositions of *M. circinelloides*.

To compare, the models were evaluated on hyperspectral images of *M. circinelloides*. Spectra of *M. circinelloides* are very challenging for scatter correction, as these fungi have a complex morphological structure and a heterogeneous chemical composition: (i) *M. circinelloides* is expected to show morphologies such as flat hyphae, swollen hyphae and yeast-like cells; (ii) the morphologies may differ in chemical composition: in oleaginous *M. circinelloides* yeast-like cells and swollen hyphae are rich in lipids, while flat hyphae are rich in cell wall components such as polysaccharides (e.g chitin and chitosan) and polyphosphates; (iii) the development of *M. circinelloides* depends on the growth conditions: under optimal growth conditions and high C/N ratio, filamentous fungi form thin cell walls and accumulate high amounts of lipids. When the C/N ratio is low, they develop thick cell walls and store lower amounts of lipids.^40,45^ In the following, we will present correction examples for spectra of oleaginous *M. circinelloides* ranging from quasi scatter-free to highly scattered spectra. We evaluate the performance of the correction approaches for whole hyperspectral images to provide a comprehensive understanding of the advantages and disadvantages of both universal and chemistry-aware scatter correction approaches.

In the case of a quasi scatter-free spectrum that does not require substantial correction, a correction algorithm should not change relative peak heights considerably. Both suggested methods, PeakLiNN and ChemLiNN, conserved the relative peak height as represented in the raw spectrum. In contrast, the DSAE method noticeably altered the relative peak heights (Figure 3A). Specifically, the lipid-related peak at 1745 cm^–1^ (associated with the –C=O bond), which is crucial for analyzing lipid production capabilities of the fungus, was reduced by approximately 50% by the DSAE method.

**Figure 3:**
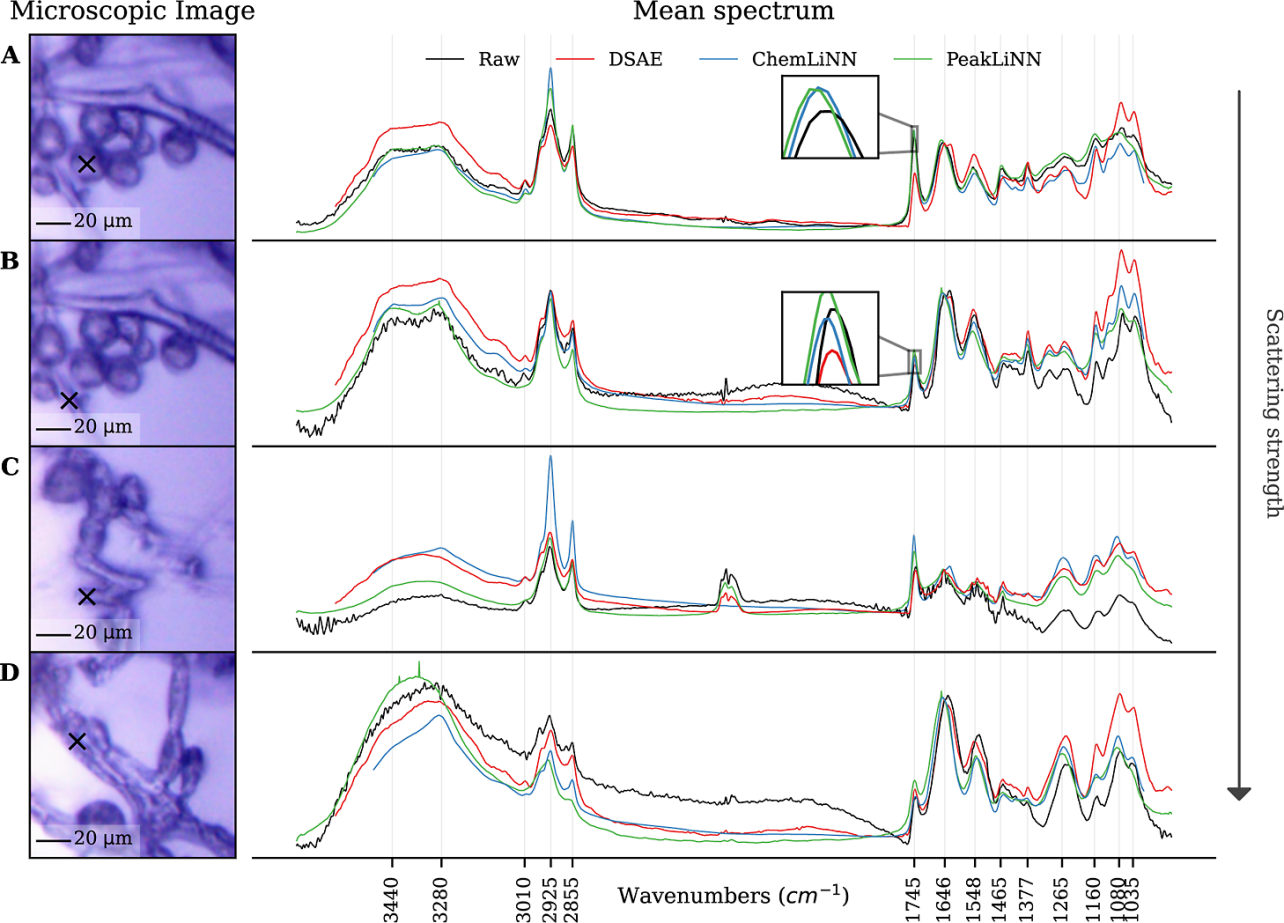
Correction of spectra taken from hyperspectral images of filamentous fungi. The raw and corrected spectra are averaged over the 3 × 3 region of (A) yeast-like cell, (B) hyphae, (C) swollen hyphae and (D) hyphae on the microscopic images. Filamentous fungi were grown (A,B) under low C/N ratio conditions leading to low lipid accumulation; and (C,D) under high C/N ratio conditions leading to high lipid accumulation. For better comparison, the spectra were normalized by the amide I peak and ordered by scattering strength. The displayed spectra show in general moderate scattering signatures. The spectral data was obtained with an agilent FTIR-FPA microscope.

The DSAE method was introduced to expedite the ME-EMSC correction process, denoise spectra, and create a method less dependent on reference spectra. However, it is important to note that DSAE is entirely dependent on the training set, where corrected spectra are obtained from the ME-EMSC method. As discussed earlier, the ME-EMSC correction converged closer to the reference spectrum representing the average chemical information on the whole cell, which may not accurately represent regions with very high or very low lipid content. This averaged reference spectrum has a lower lipid content than the actual spectra in the lipid-abundant region referred to in Figure 3A. Consequently, the DSAE was unable to handle situations with high lipid content effectively.

In contrast, the proposed neural networks, PeakLiNN and ChemLiNN, do not rely on other correction techniques and learn to make corrections directly by solving the inverse Mie scattering problem. Therefore, they are not constrained to a subset of spectra and are more robust in this regard.

For a spectrum with minor scattering features, all the methods successfully corrected Mie scattering effects (Figure 3B). They removed a small bump in the chemically inactive region (2300 cm^–1^–1800 cm^–1^); and slightly up-lifted the raw spectrum in the fingerprint region (1500 cm^–1^–900 cm^–1^). However, we observed that occasionally some spectra with broad but minor scattering features remained unchanged when the PeakLiNN model was applied (Figure S4 in Supporting Information). Interestingly, such instances occurred at the edge of spherical cell bodies. This may be due to the fact that Mie theory does not adequately explain these scattering features, or possibly because the weak and broad scattering features are interpreted as broad absorption bands. Consequently, the neural network might be using chemical features to model these scattering effects. Additionally, all the methods effectively smooth out noise in the spectra. As mentioned in the previous section, the PeakLiNN model also removed low peaks at 3010 cm^–1^ and 1035 cm^–1^, as it could not distinguish them from the noise. However, medium-sized peaks with moderate noise were preserved by the PeakLiNN model (e.g., the peak at 1548 cm^–1^ in Figure 3C). Therefore, when low peaks are important, it is beneficial to run the PeakLiNN model on low-noise spectra or to average spectra to achieve a better signal-to-noise ratio. On the other hand, the ChemLiNN model was trained on pure chemical absorbance spectra and has learned where to expect chemical peak positions. Consequently, it removes noise while preserving smaller peaks. For example, in Figure 3D, ChemLiNN model preserved the peaks at 2855 cm^–1^ and 1035 cm^–1^, whereas PeakLiNN model smoothed them.

When a raw spectrum has more pronounced scattering features, we see that all models are able to correct them, although with slight differences (Figure 3C). The PeakLiNN model tends to preserve the shape of the wide chemical peak around 3300 cm^–1^ and the peaks at 2925 cm^–1^ and 2855 cm^–1^ while the chemistry-aware methods adapt them to the chemical subspace they were conditioned to. Further, the PeakLiNN and DSAE models preserved the CO_2_ peak at 2349 cm^–1^ (ν_3_, O=C–O asymmetric stretching), while the ChemLiNN model removed it. The CO_2_ peak was not present in the training set of the ChemLiNN model, so the model has learned to always predict the absence of absorbance. This exemplifies the main pitfall of the chemistry-aware neural networks: they tend to select as a solution a spectrum from the learned chemical subspace. In other words, they tend to ignore peaks at the positions where they were absent in the training set and to create peaks at the positions where they were present in the training set irrespective of the information in a raw spectrum (Figure 4F). This behavior of the chemistry-aware neural networks can be modified by including appropriate variability in the training set, with the PeakLiNN approach taking this idea to the ultimate.

**Figure 4:**
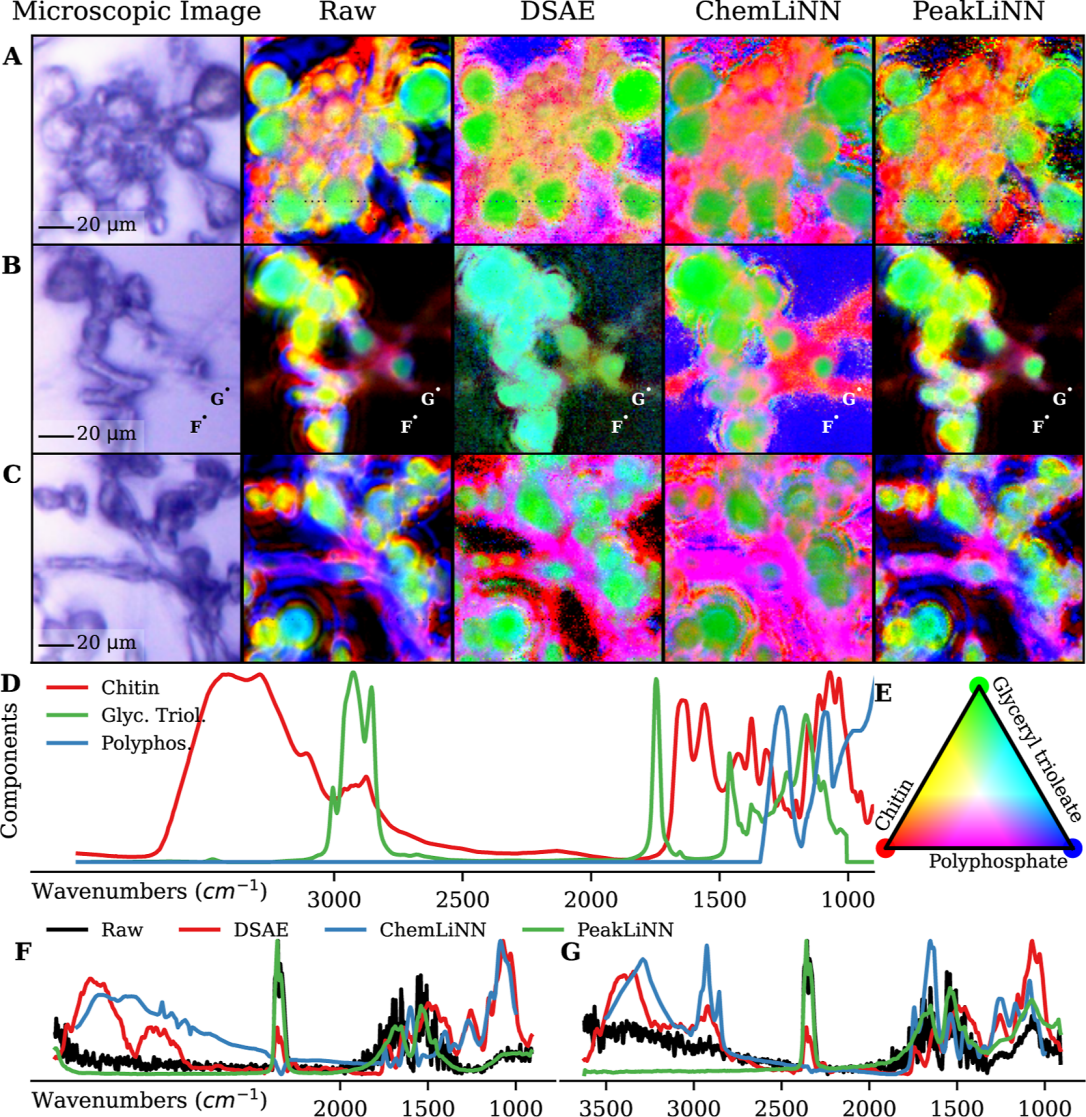
Decomposition of hyperspectral images of filamentous fungi (Mucor circenelloides, A–C) into three components (D): glycerol trioletate (yeast-like cell, green color), chitin (cell wall, red color) and polyphosphate (nutrient, blue color). The components are fitted to the raw and preprocessed (DSAE, ChemLiNN, PeakLiNN) hyperspectral images with non-negative least-squares extended with a second order polynomial for unmodeled variation. Spectra and components were min–max normalized before fitting. The regression coefficients are displayed as false color images according to the triangle legend (E). (F,G) Spectra from the correspondingly marked regions in (B) illustrating a fundamental trade-off between chemistry-dependent and chemistry-independent approaches. (F) Spectrum from a signal-free region, which appear with nonexistent fungal features for DSAE and ChemLiNN, whereas PeakLiNN did not introduce additional features. (G) Spectrum with minimal sample signal significantly amplified by DSAE and ChemLiNN, in contrast, PeakLiNN detected no signal.

Lastly, we consider the case representing a theory-practice gap, in which the irregular morphological structure of the selected fungus region is clearly distinct from a spherical shape assumed when establishing the proposed methods (Figure 3D). Inspecting the raw spectrum taken from the irregular region, we notice that the global scattering features present in the spectrum are akin to the ones predicted by the Mie theory for homogeneous spheres (see Movie 1). This observation is in agreement with the body of research concluding that the global scattering patterns (wiggles) are similar across various shapes that differ from perfect spheres.^18–21^ However, the local scattering patterns (ripples) caused by perfect spheres assumed in Mie theory occur to the lesser extent in real-life spectra, as they are caused predominantly by resonance effects such as whispering-gallery modes which are weakened in structurally irregular samples.^18^ We accounted for the suppression of ripples in the scatter simulation process by adding random constant baseline to the imaginary part of the refractive index, which had a suppressive effect on the ripples. As a result, all the established models could correct for the scattering emerged from the irregular region (Figure 3D). While the chemistry-aware models may simply produce a spectrum from the learned chemical subspace for any input (Figure 4F), thus only pretending to perform a meaningful correction, the universal PeakLiNN model does not have this option. Therefore, it is remarkable that the PeakLiNN model could produce a similar correction for an irregularly shaped sample, empirically supporting the statement that the global scattering patterns are similar for various shapes and practically showing that the scattering by a homogeneous sphere may serve as a first-order approximation for more complex structures.

### The Core Trade-off between Universal and Chemical-Aware Models—No Free Lunch Theorem

To further evaluate the performance of the proposed methods for the correction of complete hyperspectral images, we decomposed spectra of hyperspectral images of *M. circinelloides* into three components (Figure 4): chitin (a component of the fungus’ cell wall, red), glycerol trioleate (a component of fatty acid produced by *M. circinelloides*, green) and polyphosphate (stored in both, cell wall and cell interior, blue). The decomposition was done by fitting the three components and a second order polynomial (to account for the varying baseline) to the spectra in hyperspectral images with non-negative least-squares. The spectra and the components were min–max normalized before fitting. After scatter correction, as the decomposition shows, the morphology of fungi is clearly separated into lipid-rich regions corresponding to yeast-like cells and chitin-rich regions corresponding to hyphae with incorporated lipid bodies. These findings are in accordance with the overall biology of filamentous fungi. In general, all the methods are in agreement with the determination of morphology of the fungal single cells. However, in Figure 4B we see that the DSAE and ChemLiNN methods identify a region of hyphae to the right of the fungus which me marked with the letter G. The PeakLiNN method does not reveal these hyphae structures at the position G. Spectra for the position G are displayed in Figure 4G. This supports the findings that the chemistry-aware models are very sensitive to chemical information in the spectra and can produce plausible results from spectra with a very low signal-to-noise ratio, while chemistry-unaware models are in principle unable to distinguish between noise and a weak signal. In contrast, the high sensitivity of the chemistry-aware methods leads to the prediction of fungi spectra in regions surrounding sample that actually have no sample signal (Figure 4A,C). Thus, the high sensitivity complicates the precise localization of the boundary of the fungi. Even in the empty regions located far away from the fungi, where spectra are mostly consisting of noise, e.g. at the position F, the chemistry-aware models DSAE and ChemLiNN predict spectra with several false chemical features, while the chemistry-unaware model PeakLiNN handles such no-signal spectra correctly (Figure 4F). Thus, empty regions consisting of noisy spectra are identified as noise by correction with the universal model PeakLiNN. This result reflects the well-known trade-offs formalized by the No Free Lunch theorem, which asserts that optimizing an algorithm for a specific class of problems inherently limits its performance in others.^46^ In this case, optimizing a neural network to predict some particular chemical distribution (like ChemLiNN) limits its performance on spectra out of that distribution (e.g., signal-free spectra).

In summary, the universal PeakLiNN model has demonstrated a comparable correction of *M. circinelloides* spectra to the chemistry-aware models, DSAE and ChemLiNN. The PeakLiNN model effectively corrected spectra even within highly irregular morphological regions of filamentous fungi. This practical application suggests that the homogeneous sphere model employed in Mie theory can serve as a first-order approximation for more complex structures. The main challenges for the PeakLiNN model were: (i) distinguishing between noise and low peaks, which sometimes resulted in the removal of low peaks. (ii) Differentiating between absorbance and scattering features, especially at the edges of spherical bodies, occasionally leading to corrections of only noise without addressing scatter. In contrast, chemistry-aware models excel in preserving all peaks during correction, even in the presence of significant noise. However, they tend to predict spectra similar to those seen in their training set, which can result in the appearance of false chemical features and the disappearance of true chemical features. This limitation can be mitigated by including appropriate variability in the training set, an approach that the PeakLiNN model takes to the ultimate level. Thus, the chemistry-aware approach is recommended when all chemical variability is known beforehand and can be prepared in the form of scatter-free spectra which can be used for training a model based on this specific chemical variation. However, obtaining such spectra may not be feasible due to high costs and the difficulty of separating some chemical components in bulk measurements. The PeakLiNN model, in contrast, can be used without any prior knowledge about the chemical composition of the samples, without requiring a database of pure absorbance spectra, and without a training process. This potential to greatly simplify and economize scatter correction makes it more accessible and cost-effective for a wide range of samples in microspectroscopy.

### Decoding Neural Network Behaviors

To investigate how the neural network approached the correction process we conducted a sensitivity analysis using the partial derivative method^47^ (Figure 5). The sensitivity map, which is also often called saliency map, visualizes the dependence of the corrected spectrum on small perturbations in the raw spectrum, with the intensity of black color signifying the strength of this dependence. Thus, the spatial configuration of the map reveals how specific regions of the raw spectrum contribute to the corrected outcome. We averaged the sensitivity map across a thousand spectra derived from hyperspectral images of filamentous fungi of the type *M. circinelloides*.

**Figure 5:**
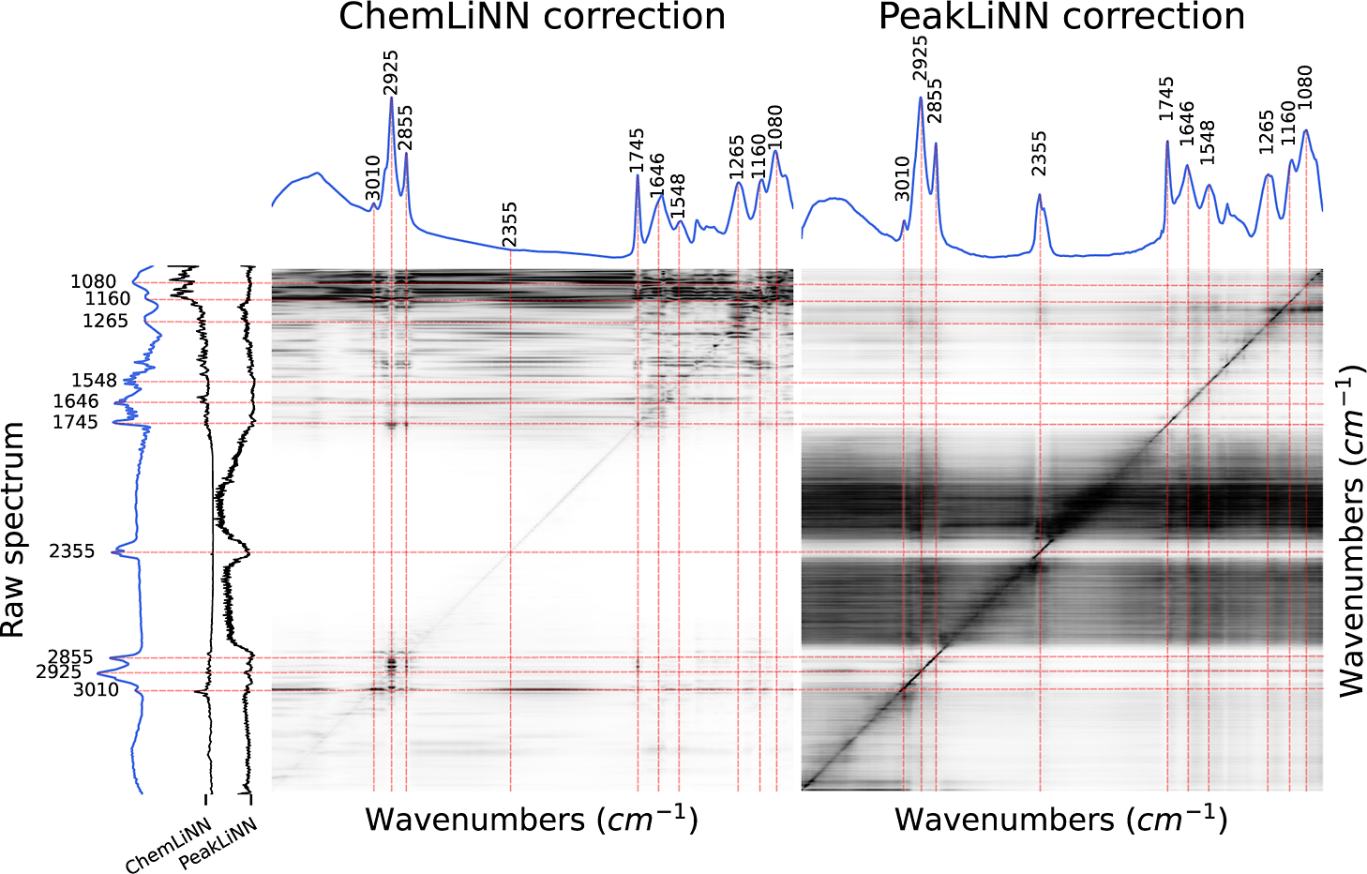
Average sensitivity of the corrected absorbance values to the raw absorbance values of trained neural networks. The sensitivity is defined as a partial derivative with respect to raw absorbance values. The sensitivity map was averaged across thousand spectra of various filamentous fungi. The black ChemLiNN and PeakLiNN lines show average importance of each wavenumber. Black color denotes strong sensitivity, while white color means no effect. For the sake of visualization, each column were standard normalized and then clipped to the 1st and 99th percentiles.

The analysis revealed that the ChemLiNN model predominantly relies on information from chemically active regions, specifically carbohydrate, protein, and lipid regions. The carbohydrate region, around 1080 cm^–1^, along with the lipid peaks at 1160 cm^–1^ and 3010 cm^–1^, significantly influence the correction process across the entire spectrum. Interestingly, while the carbohydrate region impacts the correction of the whole spectrum—evidenced by the thick horizontal black stripe on ChemLiNN’s sensitivity map (Figure 5)—it bypasses the lipid peaks at 2925 cm^–1^ and 1745 cm^–1^. The correction of these lipid peaks is predominantly influenced by other lipid-associated peaks at 2925 cm^–1^, 2855 cm^–1^, 1745 cm^–1^, and 1160 cm^–1^. Conversely, the correction of the entire spectrum by the PeakLiNN model primarily depends on the silent region between 1800 cm^–1^ and 2800 cm^–1^ and the absorbance values of each respective peak, as indicated by the diagonal black line in Figure 5. This suggests that while the ChemLiNN model utilizes less scatter-disturbed chemical regions to predict absorbance values at scatter-disturbed peaks, PeakLiNN relies on information from the actual absorbance peak of the raw spectrum it aims to correct. The correction strategy learned by the PeakLiNN model resembles the approach of utilizing silent regions for Mie scatter correction, a technique employed in classical model-driven approaches such as ME-EMSC.^28,29,31^ However, the PeakLiNN model automatically identifies silent regions and employs a data-driven approach, which may be more adaptable for complex theoretical models. Such an approach may be more suitable for complicated theoretical models.

Taken together, these results suggest that in the presence of chemical conditioning the neural network learns patterns in a chemical variability allowing to predict the whole state of the sample. Presumably this makes the ChemLiNN method robust against high levels of noise in the data. Without a chemical conditioning, the PeakLiNN neural network needs to learn about the presence and absence of chemical peaks by completely understanding the scattering features and separating them from the chemical features in the measured apparent absorbance spectrum. This learning process is more demanding than relying on correlations between chemical features within a spectrum during the learning process in order to identify chemical features and separating them from ripples and wiggles which are due to scattering.

### Quantitative Evaluation of the Neural Networks

To quantitatively estimate the degree of correction of the hyperspectral images, we calculated the variability remaining in a given data set after correction. Variability generally manifests as scatter variation and chemical variation, and a correction algorithm may reduce both. However, reducing chemical variability is often not the objective of the correction approach. We estimated the degree of variability as one minus the Pearson correlation coefficient and compared it to the raw data (Table 1). The highest variability remaining after correction was observed with the PeakLiNN model, followed by the ChemLiNN model, while the lowest variability was associated with the DSAE model. Remarkably, despite the fact that all DSAE-corrected spectra were very similar to each other, it was still possible to distinguish yeast-like cells and hyphae in the corrected image (Figure 4). It is evident that the variability left after correction is strongly influenced by the variability inherent in the training set. The DSAE model was established based on a few reference spectra representing the chemical variability of a defined set of filamentous fungi, resulting in the lowest variability. The ChemLiNN model was based on a linear combination of principal components of the presented filamentous fungi, which allowed it to produce more versatile spectra. Finally, the PeakLiNN model was based on combinations of random Gauss–Lorentzian profiles, resulting in the highest variability within the predicted spectra resulting in a maximum of chemical variability despite the fact that we observed a smoothing effect when employing the PeakLiNN correction. Therefore, shaping the variability of the training set effectively allows control over the chemical variability space of the corrected data.

**Table 1:** Spectra Variability Remaining After Scatter Correction^a^

fungi	raw	PeakLiNN	ChemLiNN	DSAE
Figure 4A	0.154	0.074	0.028	0.011
Figure 4B	0.362	0.158	0.059	0.027
Figure 4C	0.255	0.094	0.023	0.005

a Mean of pearson correlation distance (1—pearson correlation coefficient (PCC)) between each sample’s spectrum and sample’s cluster was calculated, where cluster is a mean of all sample’s spectra. Higher value reflects higher variation.

Further, we evaluated how well the models can recover relative order of the peaks and their individual amplitudes as those are known to be affected by Mie scattering (Figure S6 in Supporting Information). Evaluating on the simulated random spectra, the PeakLiNN model recovered the relative order of the peaks well (Figure S5 in Supporting Information), but the relative error of the individual peak amplitudes varied from 10% to 20%. Taking into account the exceptional complexity of the generated data (Figure S2 in Supporting Information), we consider this result to be an upper bound for a possible error in real scenarios. In contrast, the ChemLiNN model recovered individual peak heights nearly perfectly for the fungi-related generated spectra (Figure S7 in Supporting Information). Further details of the performance evaluation and discussion on generalization are provided in the Supporting Information.

### Uniqueness of the Correction

Theoretically the problem of Mie scattering correction—also known as the Inverse Mie Scattering problem—is ill-posed in a sense that it has multiple solutions. Nevertheless, we could demonstrate with a broad spectrum of examples that the PeakLiNN model is capable of performing a general correction without any assumption about sample’s underlying chemistry. Therefore, we may hypothesize that the relatively weak constraint of restricting solutions to spectral shapes that are a composition of Lorenz–Gaussian profiles without any further assumption about the chemical composition may be sufficient to make the inverse problem unique. To test this hypothesis, we developed a gradient-based algorithm that allows to find other, while not necessarily all solutions to the inverse Mie scattering problem (see details in the Supporting Information). The analysis of the found solutions confirms that the solutions near the original one indeed start to quickly develop atypical spectral features, such as fringes and sine-like broad oscillations (Figure 6) which could not be achieved with Lorenz–Gaussian profiles. Therefore, we hypothesize that if a second solution exists for the inverse Mie scattering problem in spectroscopy, then it may exhibit atypical spectral features that are unlikely to be represented by a random composition of Gauss–Lorentzian profiles.

**Figure 6:**
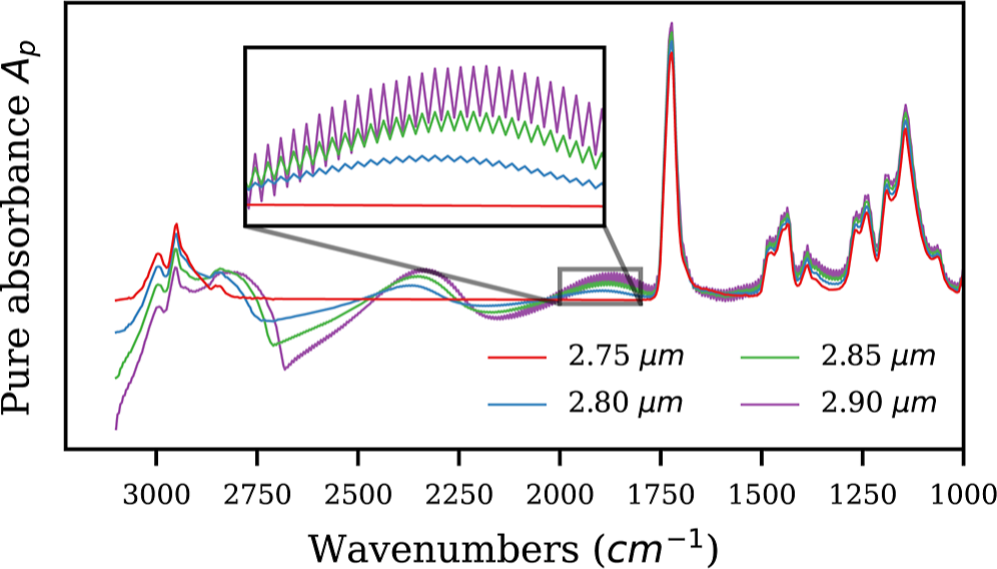
Numerical solutions for the inverse Mie scattering problem that were found in the vicinity of the pure spectrum of PMMA sphere with a radius of 2.75 μm. As the radius was gradually increased by 0.001 μm up to 3 μm, the pure spectrum was adjusted to remain consistent with a solution to the inverse Mie scattering problem. Only few pivotal spectra are displayed to avoid cluttering the figure.

Thus, the performed numerical analysis of the uniqueness of the inverse Mie scattering problem sheds a light onto why the PeakLiNN method may successfully correct Mie scattered spectra: it effectively sieves out all the solutions that do not resemble a spectrum defined as a composition of Gauss–Lorentzian profiles. This allows the PeakLiNN method to perform the chemistry-independent correction.

### Conclusion

This study reveals the possibility of a reference-free Mie scatter correction in infrared microspectroscopy. Even though we have shown that Mie scatter correction theoretically may result in several corrected pure absorbance spectra, our results suggest that only one of those solutions would be practically viable in the context of MID-IR spectroscopy, while all the rest exhibit spectral features that are nontypical for IR spectra. Without loss of universality, these nontypical features were ruled out by representing IR spectra as an arbitrary composition of Lorenz–Gaussian profiles. This condition on the shape of spectra was indirectly imposed by the data set and made the PeakLiNN model capable of a reference-free Mie scatter correction. With an exception of few spectra measured at the edges of spherical bodies using an FPA detector, the universal applicability of the model was successfully shown with real-world spectra of microplastic beads, lung cells, and filamentous fungi. Moreover, the PeakLiNN model was trained to correct spectra in a wide range of wavenumbers from 500 to 6000 cm^–1^ and with a digital spacing of 0.5–3 cm^–1^, which is covering most of practical MID-IR setups. The model is freely accessible at https://github.com/BioSpecNorway/peaklinn. Comparison with the chemistry-dependent models ME-EMSC, DSAE and ChemLiNN has shown that the versatility of the PeakLiNN model was achieved at the cost of less accurate peak amplitude predictions. The chemistry-dependent models inevitably demonstrated bias toward the given chemistry variability, which resulted in hallucinating presence or absence of false spectral features. This potentially may lead to the loss of serendipitous spectral features. For the PeakLiNN model, being chemically unconditioned helps to preserve all the spectral features that can be distinguished from noise. Analyzing sensitivity (saliency) maps we demonstrated that the ChemLiNN model ignored most of the raw spectrum and focused only on chemically relevant regions to do the correction, while the PeakLiNN model adopted the strategy of deducing scattering features relying on absorbance-free regions, which allows it to perform universally across various chemical compositions. Novelly, we have shown that Mie scatter correction is possible without any knowledge of underlying chemical composition of a sample, which has a potential to facilitate microscopic studies of biochemical samples by MID-IR spectroscopy when a reference spectrum is not readily available. We believe that our findings will inspire novel solutions in other fields were Mie scattering becomes a challenge.

## Supplementary Material





## Data Availability

The code used to generate the training set, train the PeakLiNN and the ChemLiNN models and perform evaluation, as well as the trained model weights, and data for validation will be publicly available at GitHub https://github.com/BioSpecNorway/peaklinn and Zenodo 10.5281/zenodo.15462720 upon publication of the paper. The experimental data is available upon request at Zenodo 10.5281/zenodo.17144997.

## Notes

Neural network architectures and training details (pages S2–S3), spectra simulation details (pages S4–S9), quantitative performance analysis of the PeakLiNN model (pages S10–S12), details on the uniqueness of the solution (pages S13–S16), Figures S1–S10 (PDF)
Visualization of simulated Mie scattering from a homogeneous microplastic bead composed of PMMA (AVI)

## References

[1] Bellisola G., Sorio C. (2012). Infrared Spectroscopy and Microscopy in Cancer Research and Diagnosis. Am. J. Cancer Res..

[2] Käppler A., Fischer D., Oberbeckmann S., Schernewski G., Labrenz M., Eichhorn K.-J., Voit B. (2016). Analysis of Environmental
Microplastics by Vibrational Microspectroscopy. FTIR, Raman or Both?. Anal. Bioanal. Chem..

[3] Blazhko U., Byrtusová D., Shapaval V., Kohler A., Sandt C., Zimmermann B. (2025). Submicron Infrared Spectroscopy Assessment of Single-Cell
Phenotypic Diversity in Microbial Lipid Production. Microb. Cell Fact..

[4] Perisic N., Afseth N. K., Ofstad R., Narum B., Kohler A. (2013). Characterizing
Salt Substitution in Beef Meat Processing by Vibrational Spectroscopy
and Sensory Analysis. Meat Sci..

[5] Bassan P., Kohler A., Martens H., Lee J., Byrne H. J., Dumas P., Gazi E., Brown M., Clarke N., Gardner P. (2010). Resonant Mie Scattering (RMieS) Correction
of Infrared
Spectra from Highly Scattering Biological Samples. Analyst.

[6] Zimmermann B., Bağcıoğlu M., Sandt C., Kohler A. (2015). Vibrational
microspectroscopy enables chemical characterization of single pollen
grains as well as comparative analysis of plant species based on pollen
ultrastructure. Planta.

[7] Blümel, R. ; Bağcioğlu, M. ; Lukacs, R. ; Kohler, A. Infrared Refractive Index Dispersion of Polymethyl Methacrylate Spheres from Mie Ripples in Fourier-transform Infrared Microscopy Extinction Spectra. J. Opt. Soc. Am. A, 33, 1687. 10.1364/JOSAA.33.001687. 27607489

[8] Hergert, W. ; Wriedt, T. The Mie theory: basics and applications; Springer, 2012; Vol. 169.

[9] Mohlenhoff B., Romeo M., Diem M., Wood B. R. (2005). Mie-Type Scattering
and Non-Beer-Lambert Absorption Behavior of Human Cells in Infrared
Microspectroscopy. Biophys. J..

[10] Jacques S. L. (2013). Optical
Properties of Biological Tissues: A Review. Phys. Med. Biol..

[11] Uličnỳ J. (1992). Lorenz-Mie
light scattering in cellular biology. Gen. Physiol.
Biophys..

[12] Malone M.
A., Prakash S., Heer J. M., Corwin L. D., Cilwa K. E., Coe J. V. (2010). Modifying
infrared scattering effects of single yeast
cells with plasmonic metal mesh. J. Chem. Phys..

[13] Magnussen E. A., Zimmermann B., Blazhko U., Dzurendova S., Dupuy–Galet B., Byrtusova D., Muthreich F., Tafintseva V., Liland K. H., Tøndel K. (2022). Deep learning-enabled Inference of 3D molecular absorption distribution
of biological cells from IR spectra. Commun.
Chem..

[14] Muthreich F., Magnussen E. A., Solheim J. H., Tafintseva V., Kohler A., Robin Seddon A. W., Zimmermann B. (2025). Analytical
and Experimental Solutions for Fourier Transform Infrared Microspectroscopy
Measurements of Microparticles: A Case Study on Quercus Pollen. Anal. Chim. Acta.

[15] Konevskikh T., Lukacs R., Blümel R., Ponossov A., Kohler A. (2016). Mie Scatter
Corrections in Single Cell Infrared Microspectroscopy. Faraday Discuss..

[16] Heller E., Xu K., Harris Z. B., Arbab M. H. (2025). Terahertz Mie Scattering in Tissue:
Diffuse Polarimetric Imaging and Monte Carlo Validation in Highly
Attenuating Media Models. J. Biomed. Opt..

[17] Lohner S. A., Biegert K., Nothelfer S., Hohmann A., McCormick R., Kienle A. (2021). Determining the Optical
Properties of Apple Tissue
and Their Dependence on Physiological and Morphological Characteristics
during Fruit Maturation. Part 2: Mie’s Theory. Postharvest Biol. Technol..

[18] Brandsrud M. A., Blümel R., Solheim J. H., Kohler A. (2021). The effect of deformation
of absorbing scatterers on Mie-type signatures in infrared microspectroscopy. Sci. Rep..

[19] Kong B., Brandsrud M. A., Solheim J. H., Nedrebø I., Blümel R., Kohler A. (2022). Effects of the coupling of dielectric
spherical particles on signatures in infrared microspectroscopy. Sci. Rep..

[20] Solheim J. H., Brandsrud M. A., Kong B., Banyasz A., Borondics F., Micouin G., Lossius S., Sulé-Suso J., Blümel R., Kohler A. (2023). Domes and semi-capsules as model
systems for infrared microspectroscopy of biological cells. Sci. Rep..

[21] Nousiainen T., Zubko E., Lindqvist H., Kahnert M., Tyynelä J. (2012). Comparison
of scattering by different nonspheri cal, wavelength-scale particles. J. Quant. Spectrosc. Radiat. Transfer.

[22] Tassiopoulou S., Koukiou G., Anastassopoulos V. (2024). Algorithms
in Tomography and Related
Inverse Problems—A Review. Algorithms.

[23] Bao G., Li P., Lin J., Triki F. (2015). Inverse Scattering Problems with
Multi-Frequencies. Inverse Probl..

[24] Kim T., Zhou R., Goddard L. L., Popescu G. (2016). Solving Inverse Scattering
Problems in Biological Samples by Quantitative Phase Imaging. Laser Photonics Rev..

[25] Ludlow I. K., Everitt J. (2000). Inverse Mie Problem. JOSA A.

[26] Kac M. (1966). Can One Hear
the Shape of a Drum?. Am. Math. Mon..

[27] Gordon C., Webb D. L., Wolpert S. (1992). One cannot hear the shape of a drum. Bull. Am. Math. Soc.

[28] Kohler A., Sulé-Suso J., Sockalingum G. D., Tobin M., Bahrami F., Yang Y., Pijanka J., Dumas P., Cotte M., Van Pittius D. G., Parkes G., Martens H. (2008). Estimating and Correcting
Mie Scattering in Synchrotron-Based Microscopic Fourier Transform
Infrared Spectra by Extended Multiplicative Signal Correction. Appl. Spectrosc..

[29] Solheim J. H., Gunko E., Petersen D., Großerüschkamp F., Gerwert K., Kohler A. (2019). An Open-Source
Code for Mie Extinction
Extended Multiplicative Signal Correction for Infrared Microscopy
Spectra of Cells and Tissues. J. Biophot..

[30] Magnussen E. A., Solheim J. H., Blazhko U., Tafintseva V., Tøndel K., Liland K. H., Dzurendova S., Shapaval V., Sandt C., Borondics F., Kohler A. (2020). Deep Convolutional Neural Network Recovers Pure Absorbance
Spectra from Highly Scatter-Distorted Spectra of Cells. J. Biophot..

[31] Van
Dijk T., Mayerich D., Carney P. S., Bhargava R. (2013). Recovery of absorption
spectra from Fourier transform infrared (FT-IR) microspectroscopic
measurements of intact spheres. Appl. Spectrosc..

[32] Raulf A. P., Butke J., Menzen L., Küpper C., Großerueschkamp F., Gerwert K., Mosig A. (2020). Deep Neural Networks
for the Correction of Mie Scattering in Fourier-Transformed Infrared
Spectra of Biological Samples. arXiv.

[33] Guo S., Mayerhöfer T., Pahlow S., Hübner U., Popp J., Bocklitz T. (2020). Deep Learning for ’artefact’
Removal in Infrared Spectroscopy. Analyst.

[34] Raissi M., Perdikaris P., Karniadakis G. E. (2019). Physics-Informed Neural Networks:
A Deep Learning Framework for Solving Forward and Inverse Problems
Involving Nonlinear Partial Differential Equations. J. Comput. Phys..

[35] Cuomo S., Di Cola V. S., Giampaolo F., Rozza G., Raissi M., Piccialli F. (2022). Scientific
Machine Learning Through Physics–Informed
Neural Networks: Where We Are and What’s Next. J. Sci. Comput..

[36] Krishnapriyan A., Gholami A., Zhe S., Kirby R., Mahoney M. W. (2021). Characterizing
Possible Failure Modes in Physics-Informed Neural Networks. Adv. Neural Inf. Process. Syst..

[37] Hulst, H. C. ; van de Hulst, H. C. Light scattering by small particles; Courier Corporation, 1981.

[38] Li W., Bazant M. Z., Zhu J. A. (2021). Physics-Guided
Neural Network Framework
for Elastic Plates. Comput. Methods Appl. Mech.
Eng..

[39] Stancik A. L., Brauns E. B. (2008). A simple asymmetric
lineshape for fitting infrared
absorption spectra. Vib. Spectrosc..

[40] Dzurendova S., Zimmermann B., Tafintseva V., Kohler A., Horn S. J., Shapaval V. (2020). Metal and phosphate
ions show remarkable influence
on the biomass production and lipid accumulation in oleaginous Mucor
circinelloides. J. Fungi.

[41] Martens H., Stark E. (1991). Extended multiplicative
signal correction and spectral interference
subtraction: new preprocessing methods for near infrared spectroscopy. J. Pharm. Biomed. Anal..

[42] Behnel S., Bradshaw R., Citro C., Dalcin L., Seljebotn D., Smith K. (2011). Cython: The Best of
Both Worlds. Comput. Sci.
Eng..

[43] Mätzler, C. MATLAB functions for Mie scattering and absorption. version 2, 2002.

[44] Sandt C., Dionnet Z., Toplak M., Fernandez E., Brunetto R., Borondics F. (2019). Performance
Comparison of Aperture-Less
and Confocal Infrared Microscopes. J. Spectr.
Imaging.

[45] Shapaval V., Deniset-Besseau A., Dubava D., Dzurendova S., Heitmann Solheim J., Kohler A. (2023). Multiscale spectroscopic analysis
of lipids in dimorphic and oleaginous Mucor circinelloides accommodate
sustainable targeted lipid production. Fungal
Biol. Biotechnol..

[46] Wolpert D. H., Macready W. G. (1997). No free lunch theorems
for optimization. IEEE Trans. Evol. Comput..

[47] Dimopoulos Y., Bourret P., Lek S. (1995). Use of some sensitivity
criteria
for choosing networks with good generalization ability. Neural Process. Lett..

