# Supplementary material for: A Universal Approach to Mie Scatter Correction in FTIR Analysis of Microsized Samples
Source: ACS Omega. 2025 Dec 10;10(50):61710–21. doi: 10.1021/acsomega.5c07884 (PMC12750187; doi:10.1021/acsomega.5c07884)
Supplement: Supplementary file 1 [file ao5c07884_si_001.pdf]

Supporting Information: A Universal Approach to Mie Scatter Correction in FTIR Analysis of Microsized Samples

Uladzislau Blazhko,* Eirik Magnussen, Johanne Solheim, Simona Dzurendova, Volha Shapaval, and Achim Kohler

Faculty of Sciences and Technology, Norwegian University of Life Sciences, 1430 Ås, Norway

E-mail: uladzislau.blazhko@nmbu.no

Contents

Neural networks **S-2**

Architecture S-2

Training procedure S-3

Spectra simulation **S-4**

Pure absorbance spectra simulation S-4

PeakLiNN S-4

ChemLiNN S-6

Scattering simulation S-7

Complex refractive index S-7

Extinction efficiency S-8

Absorbance values S-9

Noise augmentation S-9

Performance of the PeakLiNN model **S-10**

Uniqueness of the solution **S-13**

References **S-26**

Neural networks

Architecture

Both PeakLiNN and ChemLiNN approaches utilize a UNET-like architecture, as illustrated in Fig. S1. This architecture is comprised of seven downsampling blocks followed by seven upsampling blocks. Each downsampling block is constructed with layers of convolution, batch normalization, LeakyReLU, and a Convolution Block Attention Module (CBAM),[1] with this arrangement repeated twice within the block. The convolutional layers are characterized by a kernel size of 3, with the second convolutional layer in each sequence adopting a stride of 2 to halve the wavelength dimension. The LeakyReLU activation function is applied with a negative slope of 0.01. The CBAM layers are standardized with a reduction ratio of 16 and a kernel size of 7. The architecture is designed to double the number of filters with each subsequent block, starting from 32 filters in the initial block and culminating in 2048 filters by the seventh block. At the bottleneck stage, the frequency dimension of a spectrum, initially featuring 1408 data points, is reduced to 11 points, while the feature space expands to 2048 feature maps. The upsampling blocks mirror the structure of the downsampling blocks but employ transposed convolutions for dimensionality increase. Skip connections are strategically implemented to add the output from each strided convolution in the downsampling blocks to the input of the first batch normalization layer in the corresponding upsampling block. The network's output sequence is finalized by three convolutional layers

with 32, 32, and 1 filters, respectively. The network accepts an input comprising a spectrum and its corresponding wavelength vectors.

Training procedure

The training configuration for both models is summarized in Table S1. We implemented the models in `PyTorch Lightning`[2] and trained them using the AdamW optimizer and a ReduceLROnPlateau scheduler. Each epoch consisted of 8,192 samples generated online, trained with a batch size of 128. For the PeakLiNN model, training was run for a maximum of 10,000 epochs, and the model with the lowest training loss was selected since our objective was to have the best model being able to inverse the simulated Mie scattering as accurately as possible. For the ChemLiNN model, training was run for a maximum of 1,000 epochs as it converged to low loss values much faster.

As the loss function, we used a combination of mean squared error (MSE) and Pearson correlation, with the latter weighted by a factor of four:

$$\mathcal{L} = \text{MSE} + 4 \cdot \text{Pearson}.$$

The network inputs were spectra and their corresponding wavelengths (in μm). Each spectrum was preprocessed by normalization to the $[0, 1]$ range, followed by subtraction of its mean value.

The models were initialized with PyTorch default initialization. Random seeds were fixed via the configuration files provided in the public repository.[1] No additional regularization was used beyond the weight decay term in AdamW. Training was performed on a single NVIDIA Quadro RTX 8000 GPU and required approximately 8 hours to complete.

Implementation is available at the project repository `https://github.com/BioSpecNorway/peaklinn`.

[1] `https://github.com/BioSpecNorway/peaklinn`

Table S1: Training details for the two models.

**Parameter**	**PeakLiNN**	**ChemLiNN**
Framework	Pytorch[3]	
Training parameters		
Total epochs	10000	1000
Selected epoch	9789	624
Best loss	0.23344	0.00728
Batch size	128	
Generated samples per epoch	8192	
Loss function	MSE + 4 * Pearson correlation	
Optimizer AdamW[4]		
Learning rate	0.0001	0.000303
Weight decay	0.0001	
Scheduler ReduceLROnPlateau		
Factor	0.75	
Patience	30	
Threshold	0.005	
Factor	0.75	
Min. learning rate	1×10^{-5}	

Spectra simulation

Pure absorbance spectra simulation

In the following, we describe generation of a single spectrum for the PeakLiNN and ChemLiNN models.

PeakLiNN

For the PeakLiNN approach we first generated a floating range of wavenumbers. The range contained 1408 points, the starting point of the range varied from 400 to 3300 cm^{-1} and the end point of the range was identified depending on the resolution that varied within 0.5 - 3 cm^{-1}. Thus, the end of the generated range of wavenumbers could reach the point at ~6000 cm^{-1}. To the generated wavenumbers we added a small normally distributed random value that is resolution dependent (mean = 0, std = resolution*0.001) to avoid placing wavenumbers always on the exact grid. Further, we generated random number of asymmetric

Lorentz-Gaussian profiles within the generated range of wavenumbers as it is described in.[5] Lorentzian and Gaussian profiles were linearly combined in random proportions to obtain the Lorentz-Gaussian profile. Each Lorentz-Gaussian profile had random amplitude, position, width and skew. We distinguished sharp and wide profiles, where sharp profiles had full width at half maximum (FWHM) less than 100 cm^{-1}, while wide profiles had FWHM above 100 cm^{-1}. Asymmetry was present only in the wide profiles, while the sharp profiles exhibited zero skew. For each spectrum we generated up to 15 wide and up to 60 sharp profiles. We used such a proportion, since a typical spectrum usually has fewer number of wide peaks than sharp peaks. Furthermore, because FTIR spectra often exhibit extensive non-absorbing regions (e.g., 1800–2800 cm^{-1}), we simulated such gaps by varying their size randomly from 0% to 60% of the total wavenumber range and adjusting their position. All the simulation parameters are listed in Table S2. Thus, PeakLiNN model was trained not only on spectra representing random and unnatural chemical composition, but also on spectra with varying wavenumber ranges, so it is capable to correct raw spectra obtained from different spectrometers in different ranges and with different spacing.

Table S2: Parameters used to simulate pure absorbance spectra for the PeakLiNN model.

**Parameter**	**Value**
Wavenumbers generation	
Resolution	0.5 - 3.5 cm^{-1}
Number of points	1408
Minimal wavenumber	400 cm^{-1}
Resolution	0.5 - 3.5 cm^{-1}
Noise	$\mathcal{N}(0, Resolution * 0.001)$
Sharp Lorentz-Gaussian peaks	
Number of peaks	0 - 60
Width	5 - 100 cm^{-1}
Amplitude	0 - 1.5
Skew	0
Smooth Lorentz-Gaussian peaks	
Number of peaks	0 - 15
Width	100 - 600 cm^{-1}
Amplitude	0 - 1.5
Skew	-1/75 - 1/75

ChemLiNN

While ChemLiNN is a general approach to introduce chemical conditioning to the neural network, in paper we demonstrated it with hyperspectral images of filamentous fungus *Mucor circinelloides*. To inform the ChemLiNN model about chemical variability of the filamentous fungus, we represented the underlying chemistry by a linear combination of predefined components. The components were derived in unsupervised manner using Principal Components Analysis (PCA) of FTIR-HTS transmission spectra of homogenized filamentous fungi grown under different conditions.[6] Homogenization of the samples allowed to obtain spectra without Mie scattering effects, while varying growing conditions introduced diversity in spectra implicitly containing information about presence of various chemical components in filamentous fungi such as, for example, chitin and lipids. Notably, the obtained chemical diversity in homogenized samples allowed to describe spatial morphological variation in hyperspectral images of the filamentous fungi. Before the PCA analysis, the FTIR-HTS spectra were pre-processed by Extended Multiplicative Signal Correction (EMSC) with second derivative polynomial order.[7] The first seven principal components describing 99% of variability in the measured spectra of homogenized samples were used to represent chemical diversity of the filamentous fungi. Mixing those principal components in different proportions and adding them to the mean spectrum we simulated pure absorbance spectra of filamentous fungi. The coefficients were generated in the range from minimal to maximal score values. Since principal components by default were vector normalized, i.e. had the same area under the curve, we re-normalized them to the mean peak-to-peak value to mix them based on peak heights rather than on area under the curve. The use of the exaggerated scores values and the peak-to-peak normalized components helped to generate spectra with more pronounced variation of different components, and therefore to better simulate possible heterogeneous compositions. Since the scores were generated randomly, the generated spectra included unnatural spectra (Fig. S3).

Scattering simulation

The pure absorbance spectra, once simulated, served as the basis for generating their scattered counterparts. These simulated scattered spectra represent what could theoretically be measured in an actual experimental setup. The scattering effects were simulated using Mie theory. Thorough explanation of Mie theory can be found in.[8,9] Algorithms for Mie scattering simulation were developed in-house using Cython[10] based on Matzler's Matlab implementation.[11] In the following, we briefly state main equations and the additions we made to them. In an attempt to go beyond the rigorous simulation of scattering by an ideal spherical particle we linearly perturbed some of the theoretical variables in an empirical manner. Definitions of all the variables are explained in the table S3.

The simulation procedure of the scattered spectrum can be divided into three stages: calculation of the complex refractive index m (1), calculation of the extinction efficiency taking into account numerical aperture Q_{ext}^{NA} (2), and finally obtaining scattered absorbance spectrum A_m (3).

Complex refractive index

Complex refractive index consists out of two parts: real and imaginary. The real part represents the refractive index, which is split into the constant part n_0 and fluctuating n_{kk}. The imaginary part, also known as the attenuation coefficient, describes energy loss due to propagation in medium. According to Beer-Lambert law, the attenuation coefficient is proportional to the absorbance 1b and inversely proportional to the effective path length 1a. The effective path length represents the average path length through a sphere, which we calculated as an average chord length $\frac{\pi}{2}r$. In addition, we randomly scaled the effective path length by the parameter c_{od} for each simulated spectrum, which assumed to add variation into generated spectra making the trained model more robust towards differences in shapes. It should be noted, that while the average chord length could be represented in several ways, essentially it will be dependent on radius, and various solutions will only slightly differ by

a constant, which will not have any difference, since the effective path length is scaled by the variable parameter c_{od}. Fluctuating part of the refractive index n_{kk} is given by the Kramers-Kronig relationship 1c. Finally, the complex refractive index m is the sum of the constant and fluctuating real refractive indices, and the imaginary refractive index 1d, which was offset by random parameter $c_{n'}$ to extinguish the so called needles in Mie scattering that are typically lacking in real-world spectra due to imperfect shapes of samples.[12]

$$d_{eff} = c_{od}\frac{\pi r}{2} \tag{1a}$$
$$n' \approx \frac{A_p ln(10)\lambda}{4\pi d_{eff}} \tag{1b}$$
$$n_{kk} = Kramers - Kronig(n') = \frac{2}{\pi}P\int_0^\infty \frac{sn'(s)}{s^2 - \tilde{v}^2}ds \tag{1c}$$
$$m = n_0 + n_{kk} + i(n' + c_{n'}) \tag{1d}$$

Extinction efficiency

Mie theory describes the scattering of a plane wave by a homogeneous spherical particle, taking into account its radius r and complex refractive index m. Therefore, for each simulated pure absorbance spectrum we uniformly generated radius of spherical particle from 1 to 16 micrometers.Following the original Mie theory[8] we calculated absorbance efficiency Q_{abs} and total scattering efficiency Q^T_{sca}. Further, we took into account that infrared microscopes typically measure irradiation not only in forward direction, but also slightly off-axis, as described by numerical aperture (NA) of the objective lens. Therefore, to ensure robustness across devices with various lenses, we generated a random angle θ_{NA} from 0 to 0.9 radians. Along this angle the scattered irradiation was integrated Q^{NA}_{sca}, as described in.[9] The extinction efficiency, which describes extincted irradiation energy, was calculated as a weighted sum of the absorbance efficiency and the scattering efficiency outside of the θ_{NA} angle, which was considered to be measured by the detector. Introducing the randomly

varying parameters c_{abs} and c_{sca}, which represent a varying absorbance and scattering power, respectively, we empirically attempted to go beyond the simulation of the ideal homogeneous spherical particle.

$$Q_{abs}, Q_{sca}^{T}, Q_{sca}^{NA} = Mie(m, \lambda, r, \theta_{NA}) \tag{2a}$$

$$Q_{ext}^{NA} = c_{abs}\ Q_{abs} + c_{sca}(Q_{sca}^{T} - Q_{sca}^{NA}) \tag{2b}$$

Absorbance values

Finally, the simulated measured absorbance A_m was calculated employing the logarithmic relationship between absorbance and transmittance 3a. The transmittance can be represented as $T = 1 - \frac{g}{G}Q_{ext}$, where g is the geometrical cross section of the scatterer and G is the area of the aperture in front of the detector.[13] However, in our formula for the transmittance 3b we have used max-normalized Q_{ext}^{NA} to account for the numerical aperture and a variable parameter c_g instead of $\frac{g}{G}$ allowing to simulate various scatterer-to-aperture size ratios and thus potentially increasing robustness of the neural network towards more practical cases.

$$A_m = -log_{10}(T) \tag{3a}$$

$$T = 1 - c_g \frac{Q_{ext}^{NA}}{\max_{\tilde{\nu}}(|Q_{ext}^{NA}|)} \tag{3b}$$

Noise augmentation

Observing noise in real spectra we developed two noise augmentation techniques. The first noise technique generated high-resolution low-amplitude normally distributed noise, which then was weighted by a random second-order polynomial to simulate both noise-free and noisy regions in the raw spectra. The second noise technique generated low-resolution high-amplitude normally distributed noise, that was then weighted by a random smoothed square

signal with several alterations. Each augmentation technique was applied to the generated spectrum with a 0.5 probability, i. e. one out of four generated raw spectra were distorted by both fine-grained and coarse-grained noise augmentation.

Having the simulation procedure for pure absorbance spectra and raw spectra we could train a neural network to remove Mie scattering by giving it the simulated raw spectrum and asking to produce the corresponding simulated pure absorbance spectrum. The simulation procedure was fast enough to generate new data during the training process, so the neural network likely could never see exactly the same pair of spectra during the training.

Performance of the PeakLiNN model

Since the true absorbance spectra are rarely known in a real-world scenario, the performance was quantified for the simulated data covering various scattering parameters (Fig. S5). Often simulated data are considered to be more simplistic than real-world data. However, in our case the simulated data cover a much larger range of chemical and scattering variability as the measured data. The simulated data is not restricted for example by covariance patterns as commonly present in spectroscopic data. Thus, it is expected, that the simulated data of the PeakLiNN model covers much more chemical variability than any model that is based on pure absorbance spectra of measured data. Further, data on which the PeakLiNN model is based contains all possible ripple and wiggle structures which in general are only partially represented in measured data (Fig. S2). Taking into account such a high variability it is expected to stabilize the models. Therefore, we consider the obtained quantitative results to be a lower bound for the real-world spectra, meaning that we expect it to work better on the real-world spectra. We simulated 128 pure absorbance spectra composed out of fifteen Gauss-Lorentzian profiles placed at random positions and having various height and width. For each of those spectra we computed the corresponding Mie scattered spectra with different combinations of radius and refractive index varying within and outside the training

Table S3: Definition of variables

Theoretical variables	Description	Generation range
A_p	Simulated pure absorbance value	–
A_m	Simulated scattered (measured) absorbance value	–
λ	Wavelength	1.6 µm–25 µm
r	Radius of a simulated particle	1 µm–16 µm
d_{eff}	Effective optical path depth	–
n'	Imaginary refractive index (extinction coefficient)	–
n_0	Constant part of real refractive index	1–1.6
n_{kk}	Fluctuating part of real refractive index obtained as the Kramers-Kronig relation to n'	–
m	Complex refractive index	–
θ_{na}	Angel of numerical aperture	0 rad–0.9 rad
Mie	Matzler's algorithm to compute efficiencies according to Mie theory	–
Q_{abs}	Absorption efficiency	–
Q_{sca}^{T}	Total scattering efficiency	–
Q_{sca}^{NA}	Scattering efficiency integrated along the numerical aperture angles	–
Q_{ext}^{NA}	Extinction efficiency accounting for numerical aperture	–
Empirical parameters		
c_{od}	Optical depth multiplier	0.5–10
$c_{n'}$	Constant baseline of imaginary refractive index	0.001–0.01
c_{abs}	Absorbance power	0.05–3
c_{sca}	Scattering power	0.05–3
c_g	Parameter representing a sample-to-aperture size ratio	0.3–0.9

set ranges. Based on this we calculated a mean relative peak height error and the Spearman correlation between both the ground truth pure absorbance spectrum and the corrected spectrum. For the calculation of the metrics we used only 15 absorbance values at the simulated peak centers, excluding empty regions that might otherwise influence the metrics and result in overoptimistic values. Both metrics reveal the same pattern with respect to the scattering parameters, so we show only the Spearman correlation (Fig. S5). All other scattering parameters were randomly fixed within the training set ranges, specifically the numerical aperture angle varied within 0-0.9 radians. In average, the relative peak height error varied from 10% to 20% within the training set boundaries. The lowest error was frequently observed for peaks located in the middle of the spectrum, with the error increasing progressively towards the edges. This may indicate higher uncertainty of the solution at the edges of a spectrum. Further, the Spearman correlation varied from 0.95 to 0.85 within the training set boundaries (Fig. S5). Thus, even though the individual predicted peak heights might have a noticeable difference, their relative order was predicted overly correct. For comparison, without correction the Spearman correlation between the peaks of a simulated pure spectrum and the corresponding scattered spectrum was 0.91 for the quasi scatter-free case with a refractive index of $n_0 = 1.1$ and a radius of $r = 1\mu m$, but it quickly declined towards 0 as refractive index and radius increased introducing more scattering features (Fig. S6). This suggests that the PeakLiNN model has learned to recover the relative order of the peaks. Thus, the PeakLiNN may work well for spectra of unknown samples which are not available in a database for training. However, it may be less optimal for a subsequent regression analysis as regression models in infrared spectroscopy are often sensitive to the relative peak heights. ChemLiNN seems to be highly suitable for regression problems as it allows to restore individual peak heights almost perfectly (Fig. S7). However, as it was mentioned in the main manuscript, it tends to ignore peaks at unexpected positions (Fig. 3C in the main text).

The PeakLiNN neural network can generalize to some extent behind the training set

boundaries (Fig. S5). The learned solution appears to be more robust towards the changes in the radius rather than in refractive index. Comparing the generalization ability of the PeakLiNN and ChemLiNN, we observe that both neural networks have the ability to generalize. The ChemLiNN generalizes well along the so-called phase lag line $(n_0 - 1)r$, where n_0 is a refractive index and r is a radius (Fig. S7). The phase lag line indicates the direction along which spectra have the same global scattering pattern (wiggles), but different local scattering pattern (ripples). This shows that the ChemLiNN neural network learns well the wiggle pattern which is constant along the phase lag line $(n_0 - 1) * r$. Interestingly, the PeakLiNN neural network does not generalize in the same way along the phase lag line $(n_0 - 1) * r$. This shows that the changing ripple pattern along the phase lag line is a challenge for the PeakLiNN neural network, while the ChemLiNN model is able to deal with it. The reason for this can be understood from the sensitivity analysis of the neural networks shown in Fig. 5 in the main text. The ChemLiNN model uses information from the region below 1745cm^{-1} and in particular from the region below 1200cm^{-1} for restoring the pure absorbance in other regions. This means it uses information of a region that is to a minor degree affected from ripples and that has a feature that correlate to a feature in other spectral regions. The PeakLiNN cannot rely on such correlation within the spectrum as it did not have the opportunity to learn these correlations from real data such as the fungal data set used for training the ChemLiNN model.

Uniqueness of the solution

The inverse Mie scattering (IMS) problem is extensively studied across various scientific fields due to its relevance in characterizing particles through light scattering. The IMS problem seeks to determine the size, refractive index, or composition of spherical particles from measured scattering data such as angular intensity patterns or extinction spectra. Traditionally, the IMS problem is analysed considering a single wavelength.[14–16] In this case,

the IMS problem has multiple solutions, so given a measurement only at one wavelength, it is not always possible to distinguish between two chemically different homogeneous spheres (Fig. S8). Therefore, other sample parameters such as radius or light scattering pattern are required by single wavelength approaches to shrink the solution space. However, in spectroscopy usually a range of wavelengths is measured for the same sample. Thus, while a traditional IMS problem solution considers isolated wavelengths when solving the problem, we can take into account that the IMS problem needs to be solved for a whole wavelength range, where the same radius value is used over the whole wavelength range. Moreover, in theory, the spectrum must be continuous, which is a strong condition for the uniqueness of the inverse problem. In other words, if the IMS algorithm has learned that the inverse problem should result in a spectrum that is interconnected and looks like a spectrum, this is a constraint. A typical spectrum we expect to have a continuous, locally collinear signal. Thus, the IMS problem put in the context of infrared spectroscopy obtains additional conditions that appear to be crucial for the uniqueness of the solution. To our knowledge the uniqueness property of the IMS problem was not studied before in a such setup.

To investigate the uniqueness property of the IMS problem with all additional conditions, we devised a numerical algorithm (Fig. S9) that allows to find multiple solutions for the IMS problem, namely different pairs of pure absorbance spectrum and radius that result in the same measured spectrum according to Mie theory. The algorithm can be exemplified as follows: start with an initial pure absorbance spectrum and calculate the scattered spectrum using Mie theory for a sphere of a particular radius. Then, slightly increase the radius and find how the pure absorbance spectrum should be adjusted to preserve the same Mie scattered spectrum (Fig. 6 in the main text). Thus, by gradually increasing the radius and adjusting the pure absorbance spectrum we can obtain another numerical solution for the IMS problem.

Mathematically speaking, we introduce a function $\mathbf{A_m} \propto \mathbf{Q_{ext}} = F(r, \mathbf{A_p}, \boldsymbol{\lambda})$ that according to Mie theory calculates the measured spectrum $\mathbf{A_m}$, which is proportional to extinction

efficiency $\mathbf{Q_{ext}}$, with input radius r, the pure absorbance spectrum $\mathbf{A_p}$ and a corresponding wavelength range $\boldsymbol{\lambda}$. Note that bold font is used to represent vectors. We are then searching for two distinct pairs of radii and pure absorbance spectra $(r_1, \mathbf{A_{p1}}) \neq (r_2, \mathbf{A_{p2}})$ such that they result in the same measured spectrum according to Mie theory $\mathbf{A_{m1}} = F(r_1, \mathbf{A_{p1}}, \boldsymbol{\lambda}) = F(r_2, \mathbf{A_{p2}}, \boldsymbol{\lambda}) = \mathbf{A_{m2}}$. Since the function F is differentiable we can use the gradient-based optimization algorithms such as gradient descent or Adam to find another solutions. Starting from initial pure absorbance $\mathbf{A_{p2}} = \mathbf{A_{p1}}$ and using a slightly increased radius $r_2 = r_1 + \epsilon$, we calculate the measured spectrum $\mathbf{A_{m2}} = F(\mathbf{A_{p2}}, r_2, \lambda)$. Then we update the absorbance values of $\mathbf{A_{p2}}$ minimizing the mean squared error between the computed measured spectra: $\sum_\lambda (\mathbf{A_{m1}} - \mathbf{A_{m2}})^2$. The gradient of the function F was calculated using Pytorch's autograd engine.[17] Since the conventional gradient descent algorithm did not converge into another solution, we used the Adam algorithm that additionally accounts for a second derivative of the function.

We apply the procedure of finding another solution for the IMS problem of finding a pure absorbance spectrum to the case of infrared microspectroscopy of PMMA spheres (Fig. 6 in the main text). We start by calculating the measured spectrum A_{m1} for an initial pure absorbance spectrum of PMMA (Fig. 6 in the main text, red line) assuming that the size of the sphere is 2.75 µm. If we now try to find the same solution of the inverse problem for a sphere that is bigger by a radius difference of 0.05 µm, we find that to compensate the increase in radius the initial pure absorbance spectrum has to be noticeably adjusted at higher wavenumbers with features that are non-chemical and atypical to infrared spectroscopy (Fig. 6 in the main text, blue line). Further enlargement of the radius intensifies non-chemical features of the spectrum (Fig. 6 in the main text, green and purple lines). Firstly, broad fringes appear in order to counteract movement of the so called wiggles that are caused in the scattered spectrum by a change of radius and/or real part of the refractive index (see Movie 1). Secondly, high-frequent absorption features appear in the pure absorbance spectrum found by solving the inverse scatter problem for the slightly increased sphere. Those are

presumably counteracting changes in high frequency ripples pattern caused by the change in the radius. Further, when the radius increases by more than 0.15 µm, the calculated scattered spectrum becomes so different from the initial one (Fig. S10) that the algorithm could not compensate for this bigger change in radius with artificial pure absorbance features. Thus, the analysis revealed a limited range of numerical solutions having different pure absorbance spectra and radii that result into nearly the same scattered spectrum. Only a minority of those look like an infrared spectrum with absorption features. This indicates that the only conditioning applied for the PeakLiNN method, i.e. that the corrected spectrum should be a composition of Lorenz-Gaussian peaks, is important to make the inverse model quasi unique.

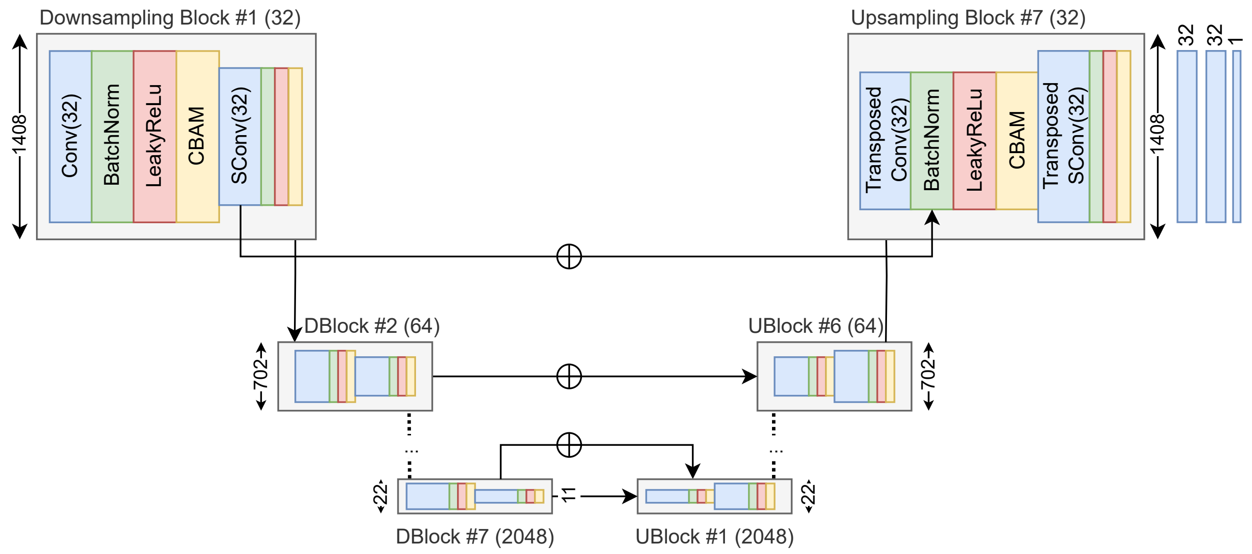

Figure S1: The UNET-like architecture of PeakLiNN and FungLiNN models, comprised out of seven consecutive downsampling and upsampling blocks.

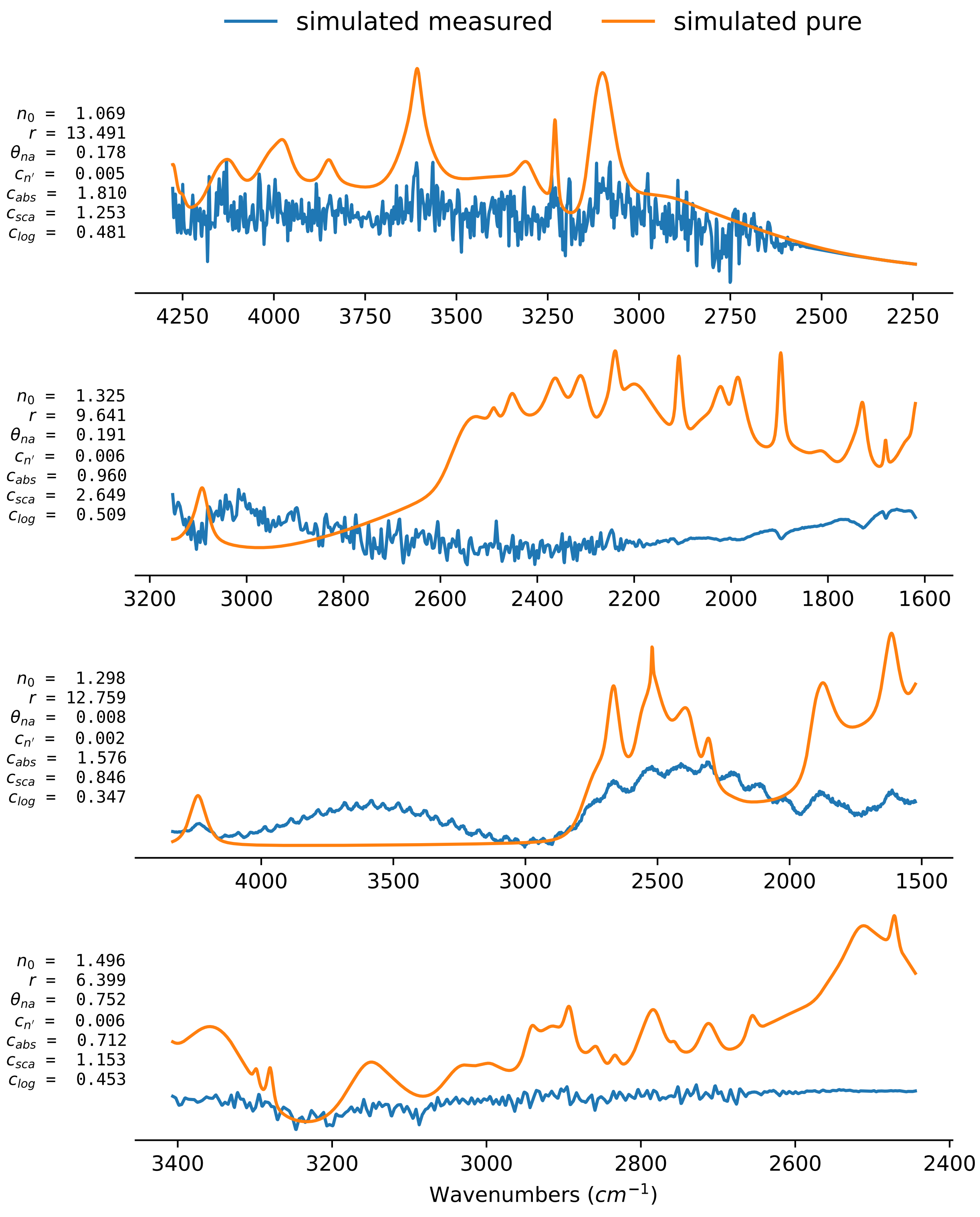

Figure S2: Difficult-to-correct examples of simulated pure and simulated measured spectra used for training the PeakLiNN model.

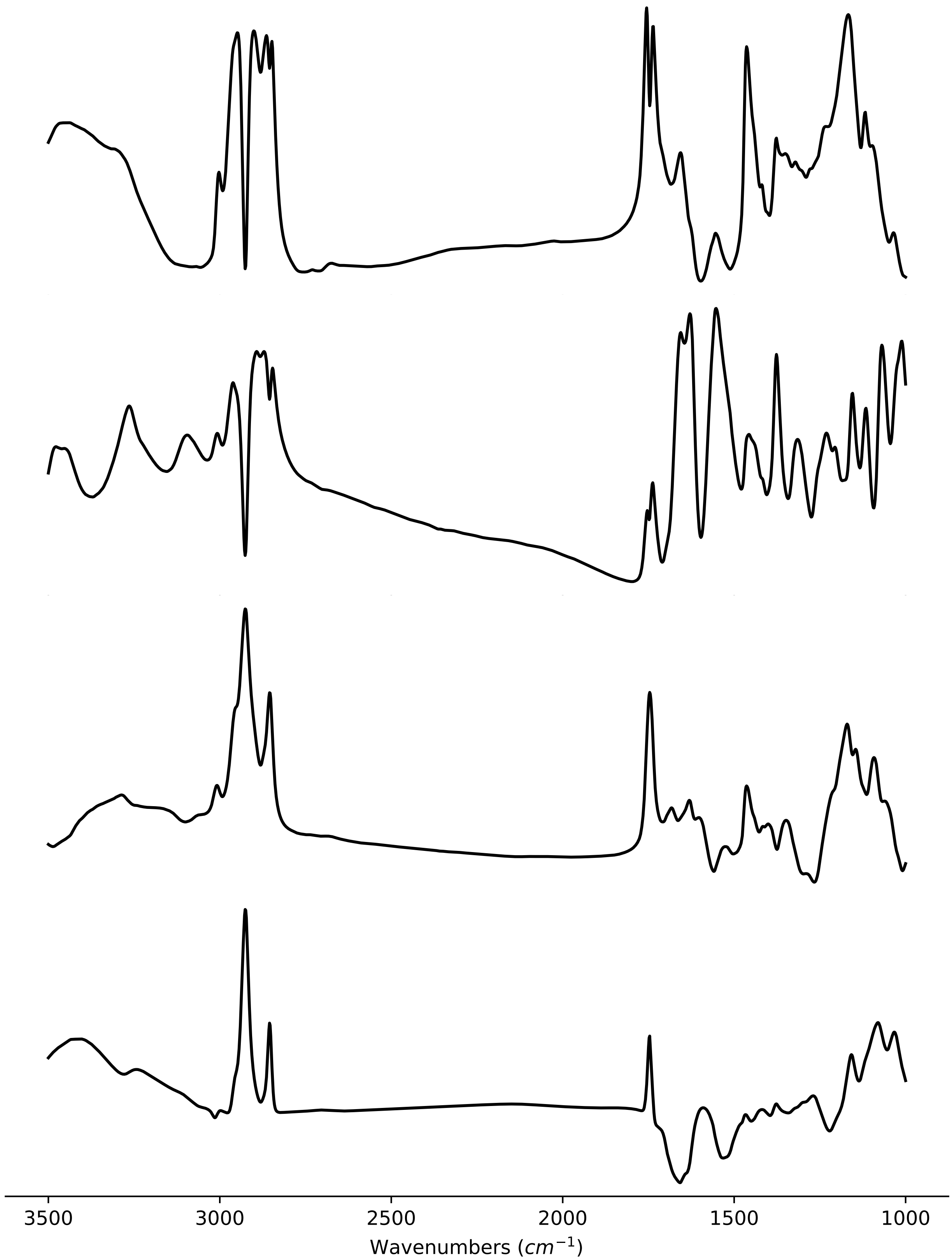

Figure S3: Examples of unnatural pure simulated spectra used to train the ChemLiNN model.

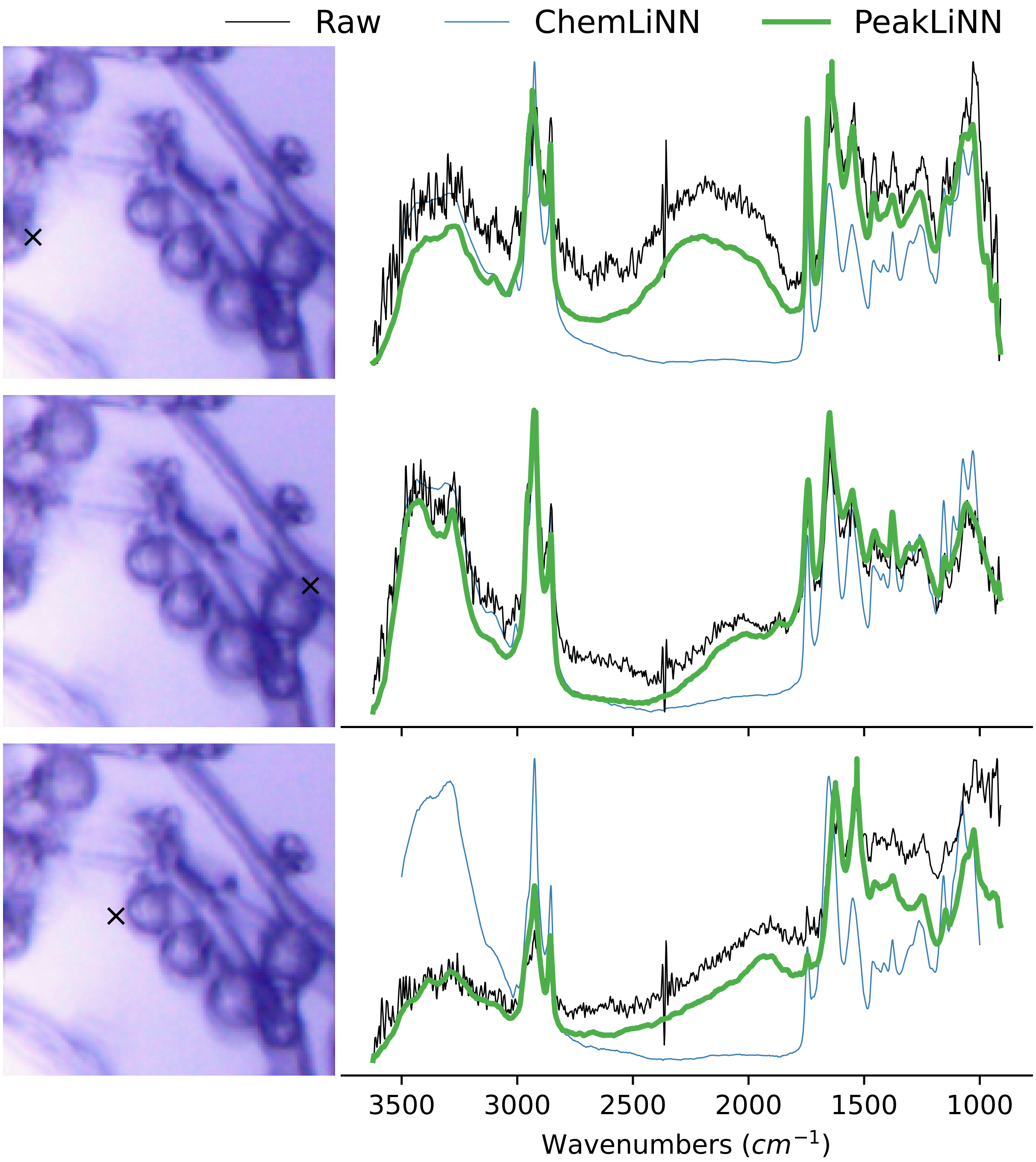

Figure S4: Occasional examples of spectra correction at the edges of spherical cell bodies, when the PeakLiNN model could not recognize scattering patterns.

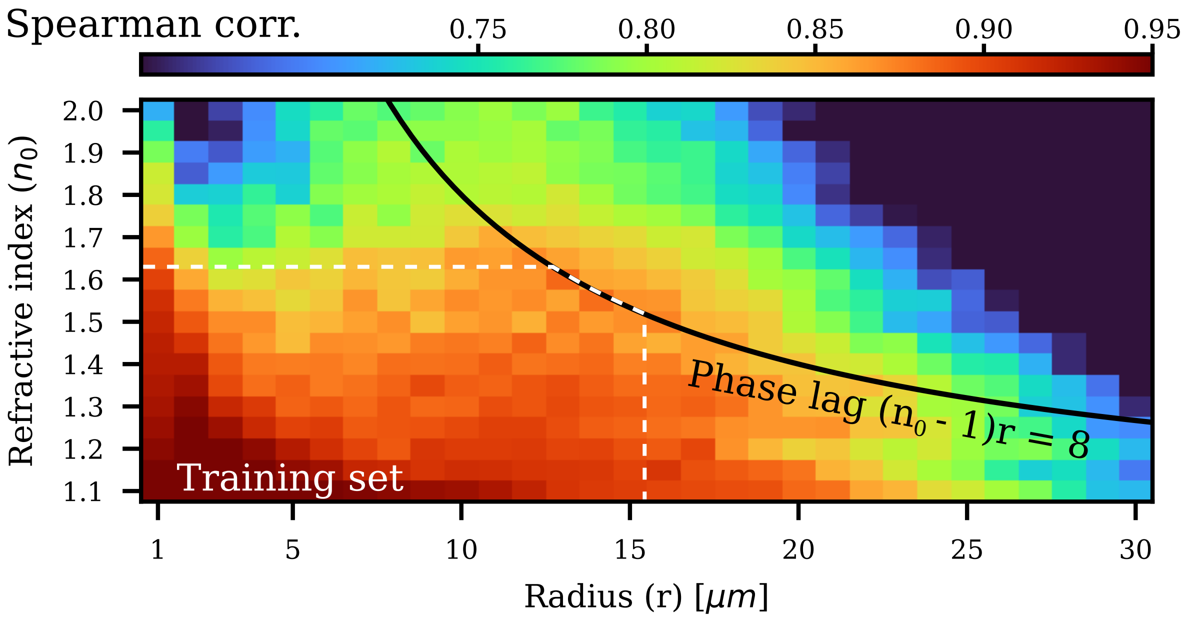

Figure S5: Average performance of the PeakLiNN method on simulated spectra with varying scattering parameters (radius and refractive index) within and outside the training set boundaries (white). The plot utilizes the Spearman correlation between 15 peak heights from both simulated pure absorbance spectra and corrected spectra. Each pixel indicates the averaged correlation over 128 simulated samples, sharing the same set of pure absorbance spectra composed out of 15 random Gauss-Lorentzian peaks but with varying measured spectra simulated per the corresponding radius and refractive index and randomized other parameters. The Spearman correlation reflects the ability of the method to maintain the relative ordering of peak heights, irrespective of their absolute values. In other words, a high correlation indicates successful correction, where the highest peak remains the highest, the second highest stays second highest, and so forth, despite the actual differences in their heights. The size factor line shows direction along which the simulated Mie scattering has the same global pattern (wiggles), but varying local patterns (ripples).

s

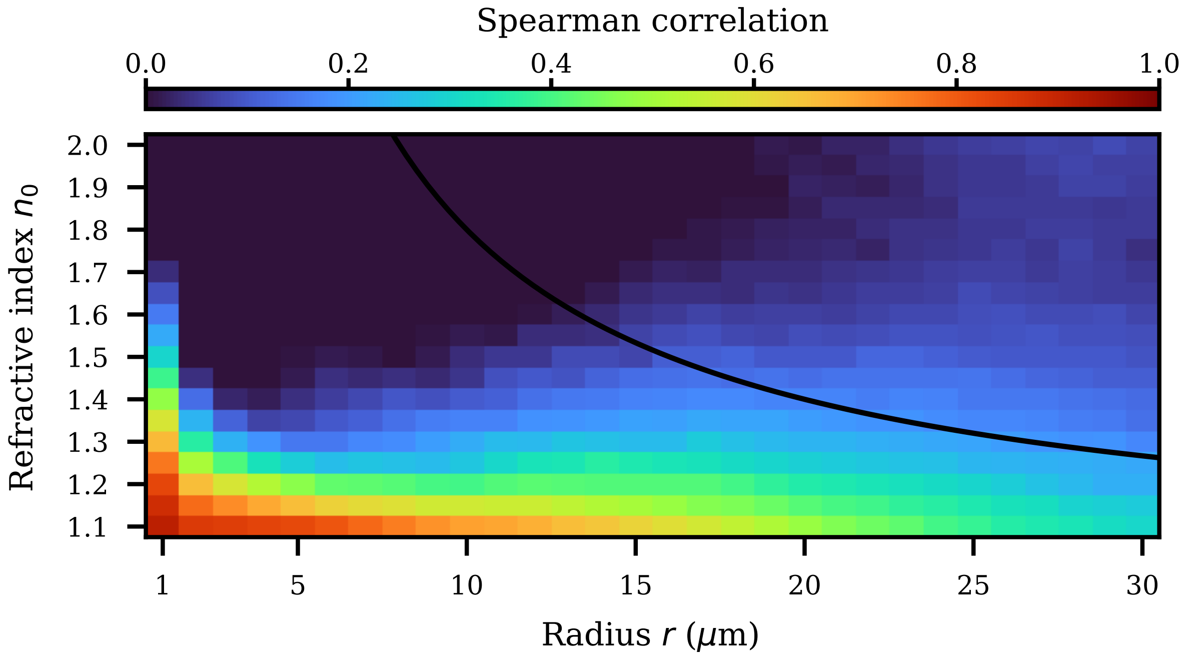

Figure S6: Spearman correlation of the simulated Mie-scattered spectrum with its pure spectrum. This shows that Mie scattering increasingly disrupts relative ordering of the peaks in the pure spectrum with increase of the scattering features.

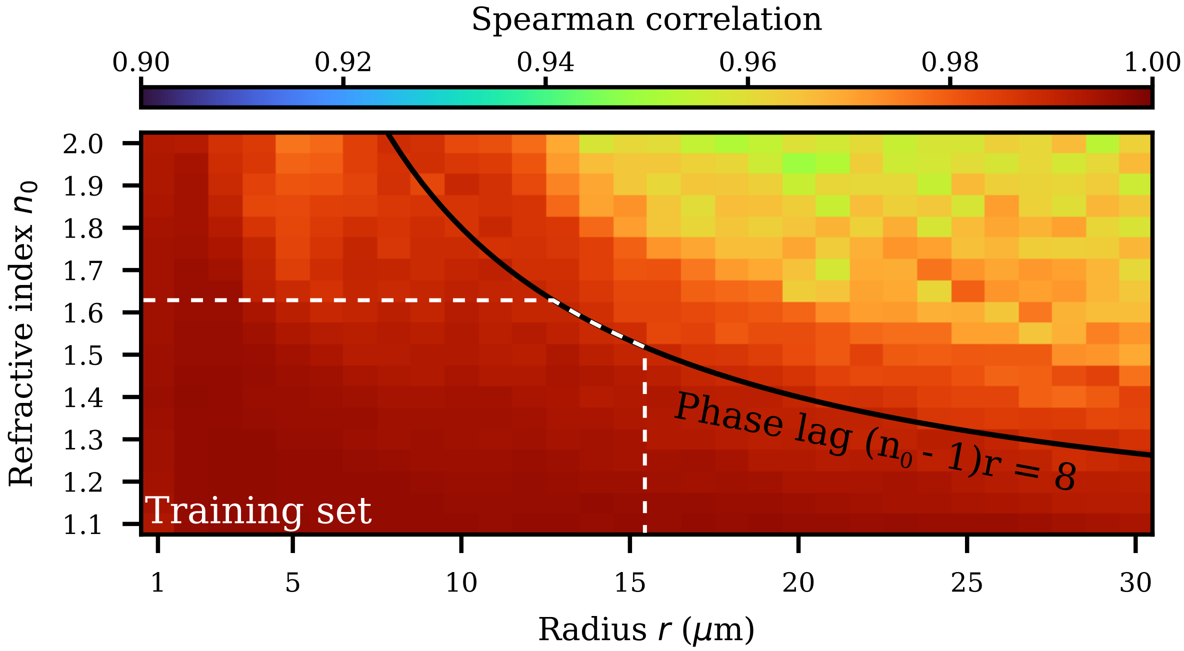

Figure S7: Spearman correlation of the ChemLiNN correction. All the map is higher than 0.95, what indicates that the correction of the ChemLiNN model is very robust and accurate on and behind its training set.

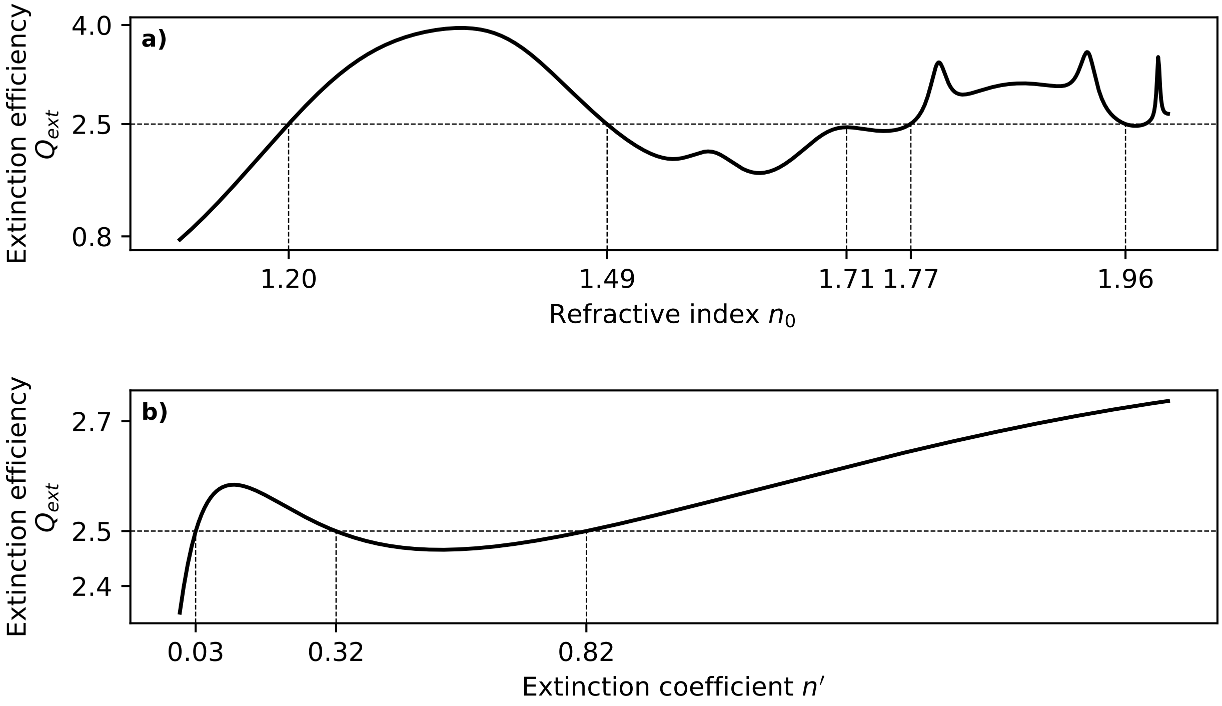

Figure S8: Non-uniqueness of the inverse Mie scattering problem for a single wavenumber. Extinction efficiency Q_{ext} is calculated for homogeneous sphere with the radius of 5 μm across (a) refractive indices from 1 to 2 and extinction coefficient $n' = 0$; (b) refractive index $n_0 = 1.5$ and extinction coefficient n' from 0 to 2. The extinction efficiency is taking the same value of 2.5 several times for different parameters, which shows its non-uniqueness with respect to its optical and chemical properties.

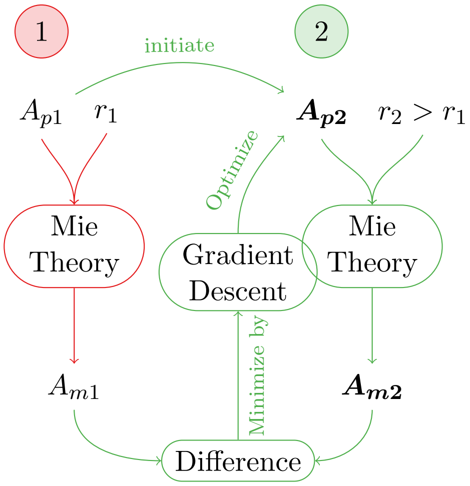

Figure S9: Algorithm of finding another solution for Mie scattering problem. Variables in bold change their values during iterations. First, we define a pure spectrum A_{p1} and radius r_1 and then, using Mie theory, calculate the measured spectrum A_{m1}. The pair (A_{p1}, r_1) is by definition the first solution to the inverse Mie scattering problem. In the second step, we are numerically finding another solution. We initialize the pure spectrum A_{p2} with A_{p1}, while slightly increase radius $r_2 = r_1 + \epsilon$ of the particle. Then, we calculate a second measured spectrum A_{m2}, which will be slightly different from the A_{m1} due to the increased radius. The idea is to compensate the difference caused by increase of radius adjusting only the pure spectrum. So we minimize the Pearson's distance between A_{m2} and original A_{m1} optimizing the A_{p2} spectrum. Since the Mie Theory formalism is differentiable we employed the gradient descent algorithm Adam. Once the optimization is done, we have found another numerical solution: A_{p2} spectrum with radius $r_2 > r_1$.

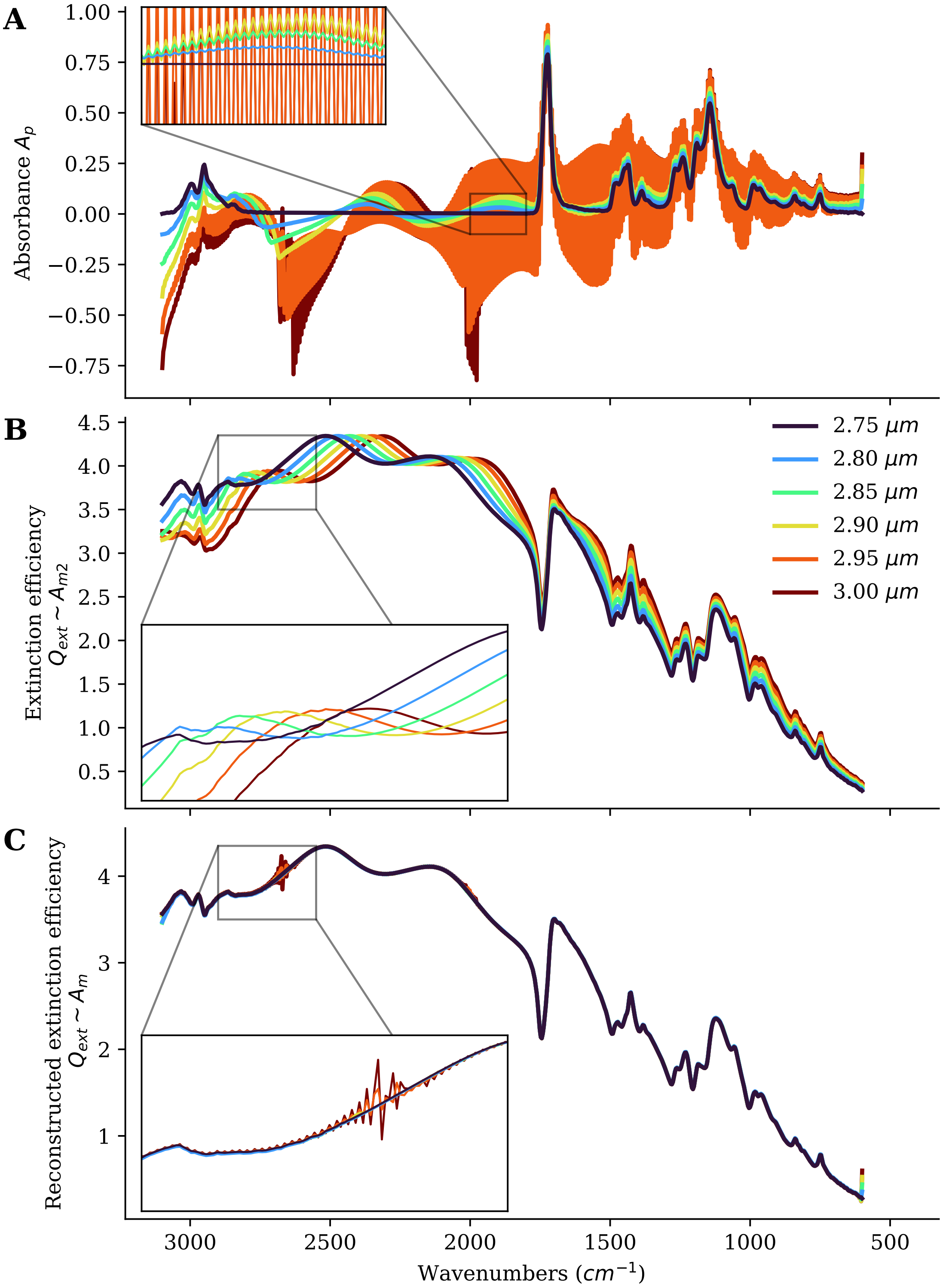

Figure S10: The results of applying the numerical algorithm to find other solutions to the inverse Mie scattering problem that was explained in the text of main manuscript. (A) Found numerical solutions A_p to the inverse Mie scattering problem. (B) Simulated measured spectra using the original pure spectrum of PMMA bead and varying radii. (C) The closest acquired simulated measured spectra after optimization of the pure absorbance spectrum A_p minimizing residuals between Q_{m1} and Q_{m2}.

References

(1) Woo, S.; Park, J.; Lee, J.-Y.; Kweon, I. S. Cbam: Convolutional block attention module. Proceedings of the European conference on computer vision (ECCV). 2018; pp 3–19.

(2) Falcon, W.; The PyTorch Lightning team, PyTorch Lightning. 2019; `https://github.com/Lightning-AI/lightning`.

(3) Paszke, A.; Gross, S.; Massa, F.; Lerer, A.; Bradbury, J.; Chanan, G.; Killeen, T.; Lin, Z.; Gimelshein, N.; Antiga, L., et al. Pytorch: An imperative style, high-performance deep learning library. *Advances in neural information processing systems* **2019**, *32*.

(4) Loshchilov, I.; Hutter, F. Decoupled weight decay regularization. *arXiv preprint arXiv:1711.05101* **2017**,

(5) Stancik, A. L.; Brauns, E. B. A Simple Asymmetric Lineshape for Fitting Infrared Absorption Spectra. *47*, 66–69.

(6) Dzurendova, S.; Zimmermann, B.; Tafintseva, V.; Kohler, A.; Horn, S. J.; Shapaval, V. Metal and phosphate ions show remarkable influence on the biomass production and lipid accumulation in oleaginous Mucor circinelloides. *Journal of Fungi* **2020**, *6*, 260.

(7) Martens, H.; Stark, E. Extended multiplicative signal correction and spectral interference subtraction: new preprocessing methods for near infrared spectroscopy. *Journal of pharmaceutical and biomedical analysis* **1991**, *9*, 625–635.

(8) Hulst, H. C.; van de Hulst, H. C. *Light scattering by small particles*; Courier Corporation, 1981.

(9) Konevskikh, T.; Lukacs, R.; Blümel, R.; Ponossov, A.; Kohler, A. Mie Scatter Corrections in Single Cell Infrared Microspectroscopy. *Faraday Discussions 187*, 235–257.

(10) Behnel, S.; Bradshaw, R.; Citro, C.; Dalcin, L.; Seljebotn, D.; Smith, K. Cython: The Best of Both Worlds. *Computing in Science Engineering* **2011**, *13*, 31 –39.

(11) Mätzler, C. MATLAB functions for Mie scattering and absorption, version 2. 2002.

(12) Brandsrud, M. A.; Blümel, R.; Solheim, J. H.; Kohler, A. The effect of deformation of absorbing scatterers on Mie-type signatures in infrared microspectroscopy. *Scientific Reports* **2021**, *11*, 4675.

(13) Lukacs, R.; Blümel, R.; Zimmerman, B.; Bağcıoğlu, M.; Kohler, A. Recovery of absorbance spectra of micrometer-sized biological and inanimate particles. *Analyst* **2015**, *140*, 3273–3284.

(14) Ludlow, I. K.; Everitt, J. Inverse Mie Problem. *JOSA A, Vol. 17, Issue 12, pp. 2229-2235* **2000**, *17*, 2229–2235.

(15) Romanov, A. V.; Konokhova, A. I.; Yastrebova, E. S.; Gilev, K. V.; Strokotov, D. I.; Chernyshev, A. V.; Maltsev, V. P.; Yurkin, M. A. Spectral solution of the inverse Mie problem. *Journal of Quantitative Spectroscopy and Radiative Transfer* **2017**, *200*, 280–294.

(16) Sumlin, B. J.; Heinson, W. R.; Chakrabarty, R. K. Retrieving the aerosol complex refractive index using PyMieScatt: A Mie computational package with visualization capabilities. *Journal of Quantitative Spectroscopy and Radiative Transfer* **2018**, *205*, 127–134.

(17) Paszke, A.; Gross, S.; Massa, F.; Lerer, A.; Bradbury, J.; Chanan, G.; Killeen, T.; Lin, Z.; Gimelshein, N.; Antiga, L.; Desmaison, A.; Kopf, A.; Yang, E.; DeVito, Z.; Raison, M.; Tejani, A.; Chilamkurthy, S.; Steiner, B.; Fang, L.; Bai, J.; Chintala, S. *Advances in Neural Information Processing Systems 32*; Curran Associates, Inc., 2019; pp 8024–8035.
